# Natural compounds targeting inflammatory signaling and cell adhesion molecules in ischemic acute kidney injury

**DOI:** 10.1007/s12272-026-01601-4

**Published:** 2026-04-26

**Authors:** Sally A. Fahim, Ahmed M. El-Dessouki, Nada Osama, Sherif S. Abdel Mageed, Mahrous H. Mahrous, Reham A. Mohammed, Ahmed S. Kamel, Kareem Abdou, Joon Hyung Yeo, Riham A. El-Shiekh, Nehal I. Rizk

**Affiliations:** 1 Department of Biochemistry, School of Pharmacy, Newgiza University (NGU), Km 22 Cairo-Alexandria Desert Road, Newgiza, 12577 Giza Egypt; 2 Pharmacology and Toxicology Department, Faculty of Pharmacy, Ahram Canadian University, 6th of October City, 12566 Giza Egypt; 3 Biochemistry Department, Faculty of Pharmacy, Menoufia University, Gamal Abd El Nasr St., Shibin Elkom, 32511 Menoufia Egypt; 4 Pharmacology Department, Faculty of Pharmacy, Badr University in Cairo (BUC), Badr City , 11829 Cairo Egypt; 5 Department of Pharmacognosy, Faculty of Pharmacy, Delta University for Science and Technology, Dakhliya, Egypt; 6 Department of Pharmacology and Toxicology, Faculty of Pharmacy, Cairo University, Kasr El-Aini Street, Cairo, 11562 Egypt; 7 Department of Pharmacy Practice, Faculty of Pharmacy and Drug Technology, Egyptian Chinese University, Cairo, Egypt; 8 Department of Pharmacology and Toxicology, Faculty of Pharmacy and Drug Technology, Egyptian Chinese University, Cairo, Egypt; 9 College of Pharmacy, Al-Ain University, Abu Dhabi, UAE; 10 College of Medicine, Chung-Ang University Gwangmyeong Hospital, Gwangmyeong, 14353 Republic of Korea; 11 Pharmacognosy Department, College of Pharmacy, Cairo University, Cairo, 11562 Egypt; 12 Department of Biochemistry, Faculty of Pharmacy and Drug Technology, Egyptian Chinese University, Cairo, 11786 Egypt

**Keywords:** Ischemic acute kidney injury, Inflammatory signaling pathways, Cell adhesion molecules, Natural compounds, Renal microcirculation, Nanocarrier-based therapy

## Abstract

Ischemic acute kidney injury (AKI) remains a major clinical challenge, characterized by high morbidity, mortality, and a substantial risk of progression to chronic kidney disease. Accumulating evidence indicates that ischemic AKI is not merely a transient hemodynamic disorder but a complex, biologically orchestrated process driven by microvascular dysfunction, innate immune activation, inflammatory signaling, and maladaptive tissue repair. Despite advances in supportive care, effective disease-modifying therapies are still lacking. Recent studies have highlighted that key signaling pathways, including Toll-like receptor/nuclear factor-κB (TLR/NF-κB), Janus kinase/signal transducer and activator of transcription (JAK/STAT), purinergic P2X7 receptor–inflammasome signaling, heat-shock protein–mediated stress responses, and phosphoinositide 3-kinase/Akt/mammalian target of rapamycin (PI3K/Akt/mTOR) cascades, govern the initiation, amplification, and resolution of ischemic renal injury. These pathways converge on downstream cellular effectors such as cell adhesion molecules (CAMs), which orchestrate leukocyte recruitment, endothelial-epithelial interactions, and spatial propagation of inflammation within the renal microvasculature. Natural compounds have emerged as promising therapeutic candidates for ischemic AKI due to their pleiotropic pharmacological properties and ability to modulate multiple pathogenic signaling networks simultaneously. A growing body of experimental evidence demonstrates that polyphenols, glycosides, saponins, and related phytochemicals attenuate ischemic renal injury by suppressing inflammatory signaling, reducing CAM expression, preserving microcirculatory integrity, and promoting adaptive repair. Furthermore, advances in nanocarrier-based delivery systems have substantially enhanced the translational potential of these compounds by improving bioavailability, renal targeting, and pathway-specific modulation. In this review, we provide a comprehensive, signaling-centered analysis of ischemic AKI pathogenesis and systematically map natural compounds to their molecular targets and downstream inflammatory effectors. By integrating mechanistic insights with emerging nanotherapeutic strategies, this work offers a structured framework for the rational development of multi-target, mechanism-based interventions for ischemic AKI. It highlights key challenges and future directions for clinical translation.

**Supplementary Information:**

The online version contains supplementary material available at 10.1007/s12272-026-01601-4.

## Introduction

**Fig. 1:**
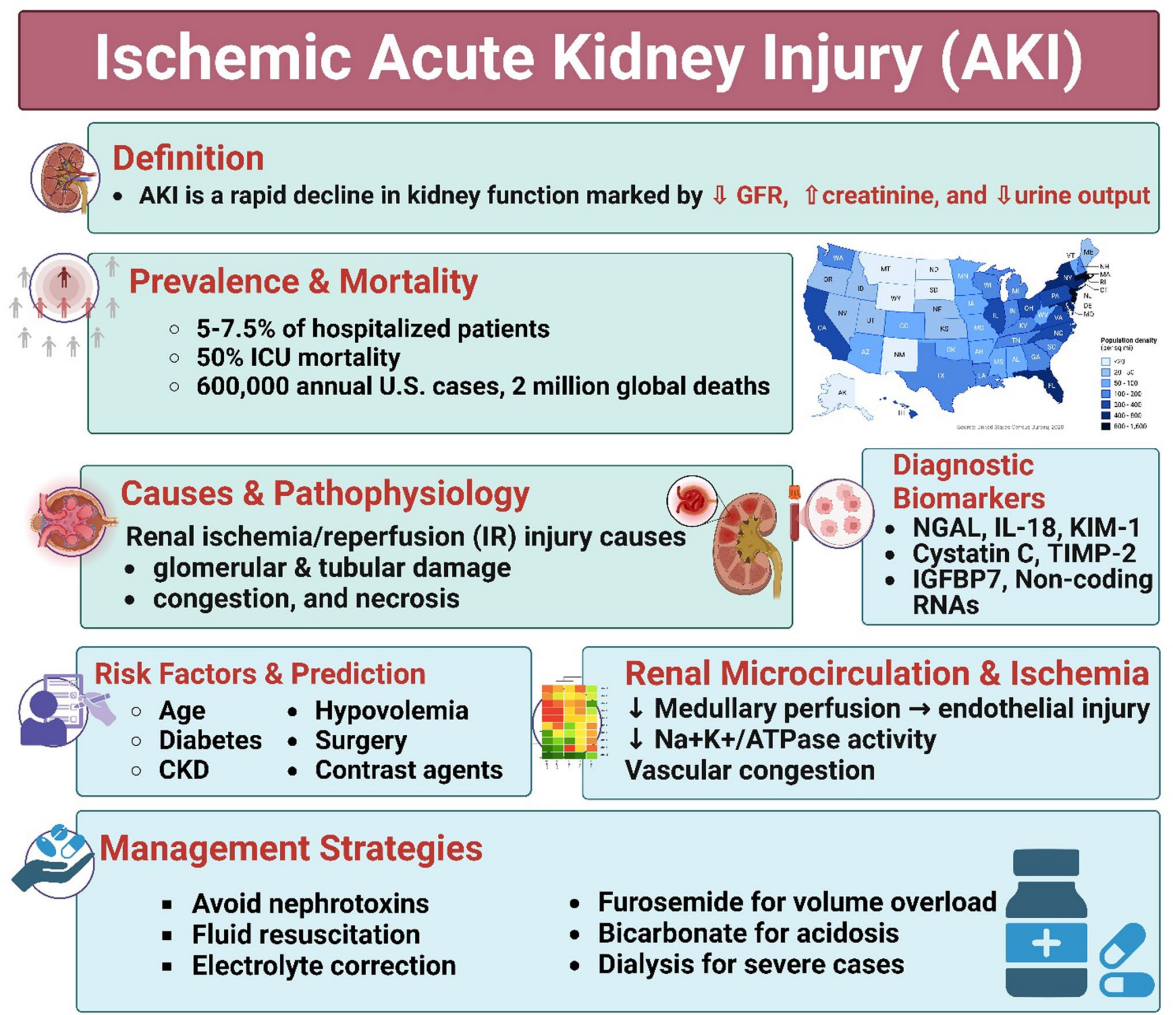
Illustrates a summary of ischemic acute kidney injury (AKI), including definition, prevalence, pathophysiology, risk factors, biomarkers, and management strategies

Acute kidney injury (AKI) is a heterogeneous clinical syndrome characterized by an abrupt decline in renal function and remains a major contributor to morbidity and mortality worldwide. AKI affects approximately 20% of hospitalized patients, with a particularly high incidence among critically ill, surgical, and elderly populations (Gaut and Liapis 2021; Pickkers et al. 2021). Importantly, AKI is no longer considered a fully reversible condition; even transient episodes significantly increase the risk of chronic kidney disease (CKD), cardiovascular complications, and long-term mortality (Kellum et al. 2021; Ostermann et al. 2024) (Fig. 1).

From a pathophysiological standpoint, AKI encompasses a complex spectrum of renal injury involving tubular epithelial cell death, endothelial dysfunction, microvascular rarefaction, and maladaptive inflammatory responses. Ischemia–reperfusion (I/R) injury represents one of the most common and clinically relevant causes of AKI, particularly in the settings of sepsis, major surgery, and organ transplantation (Mehta et al. 2004; Peng et al. 2023). At the molecular level, ischemic AKI is driven by excessive oxidative stress, mitochondrial dysfunction, and activation of innate immune signaling cascades, including Toll-like receptor (TLR)/NF-κB, JAK/STAT, PI3K/Akt/mTOR, and Sonic Hedgehog (Shh) pathways (Bai et al. 2016). These signaling networks orchestrate cytokine release, leukocyte recruitment, endothelial activation, apoptosis, and fibrotic remodeling, ultimately contributing to sustained renal damage and impaired recovery (Wang et al. 2020b,[Bibr CR216][Bibr CR217]; Liu et al. 2024).

**Fig. 2:**
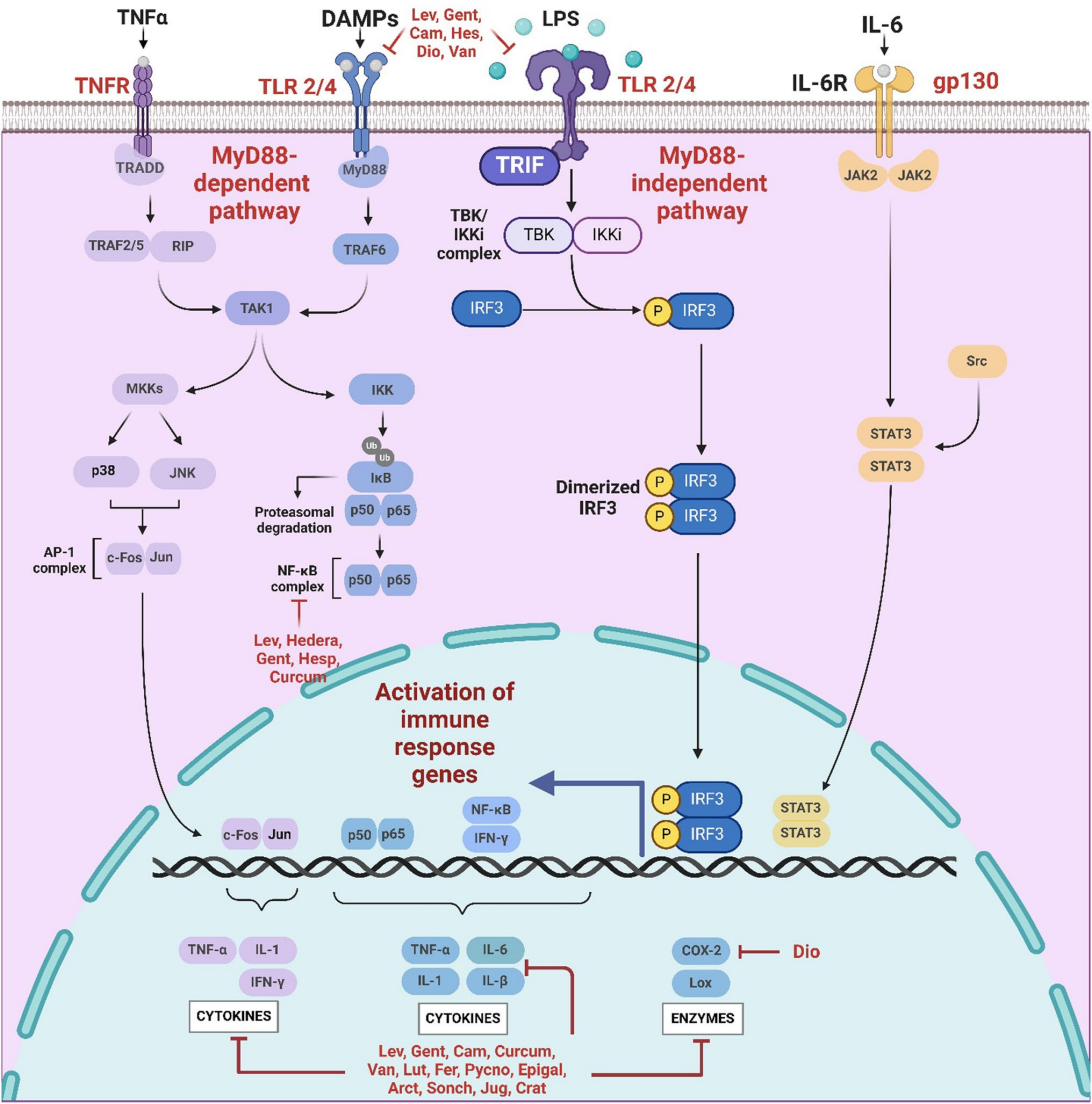
Depicts the inflammatory signaling pathways involved in renal ischemia/reperfusion injury (IRI), highlighting MyD88-dependent and -independent TLR pathways, NF-κB and IRF3 activation, cytokine release, and the modulatory effects of natural compounds. Levistolide (Lev), Gentiopicroside (Gent), Camellia oil (Cam), Hesperidin (Hesp), Dioscin (Dio), Vanillin (Van), Luteolin (Lut), Ferulic acid (Fer), Pycnogenol (Pycno), Epigallocatechin gallate (Epigal), Arctigenin (Arct), Sonchus oleraceus (Sonch), Juglans mollis (Jug), and Crateva nurvala (Crat)

Clinically, AKI manifests across a wide spectrum, ranging from subtle elevations in serum creatinine to severe multiorgan failure requiring renal replacement therapy (Mercado et al. 2019). Although emerging biomarkers such as neutrophil gelatinase-associated lipocalin (NGAL), kidney injury molecule-1 (KIM-1), cystatin C, and cell-cycle arrest markers have improved early diagnosis and risk stratification (Hu et al. 2017; Ortega and Heung 2018; Yi et al. 2021), therapeutic options remain largely supportive. Current management strategies primarily focus on hemodynamic optimization, avoidance of nephrotoxins, and dialysis in advanced cases. However, these approaches do not directly target the underlying molecular mechanisms of injury, nor do they reliably prevent progression to CKD or end-stage renal disease (ESRD) (Gaut and Liapis 2021; Yoon et al. 2022). To date, no pharmacological agent has demonstrated consistent disease-modifying efficacy in AKI, highlighting a critical unmet therapeutic need.

In this context, natural compounds have emerged as promising candidates for AKI management due to their pleiotropic pharmacological properties and generally favorable safety profiles. Accumulating experimental evidence suggests that polyphenols, polysaccharides, terpenes, sterols, and other bioactive natural products can attenuate ischemic and toxic renal injury by modulating oxidative stress, inflammation, apoptosis, and autophagy (Alhusaini et al. 2019; Kang et al. 2021; Ali et al. 2022; Alrumaihi et al. 2024). Notably, these compounds interact with multiple key pathogenic pathways implicated in AKI, including cell adhesion molecules (CAMs), Shh signaling, JAK/STAT cascades, purinergic P2X7 receptors, heat-shock proteins (Hsps), and PI3K/Akt/mTOR signaling networks. Through these mechanisms, natural products may preserve microvascular integrity, suppress maladaptive immune activation, and promote renal repair (Zhang et al. 2024a; Peng et al. 2025).

**Fig. 3:**
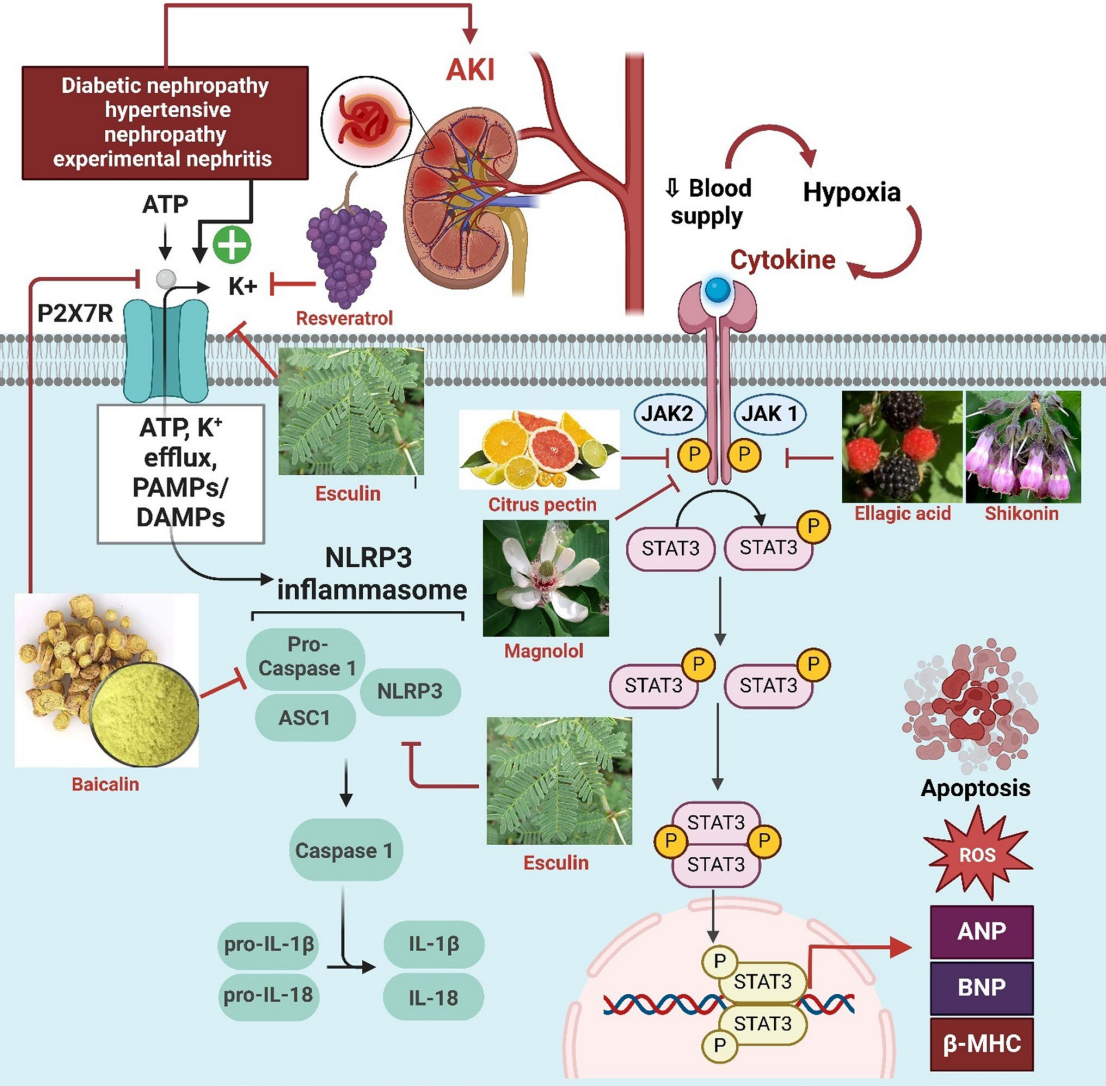
Illustrates a schematic representation of inflammatory pathways in acute kidney injury (AKI), highlighting the role of ATP-P2X7R signaling, NLRP3 inflammasome activation, and JAK/STAT3 signaling in promoting cytokine release, oxidative stress, and apoptosis. The diagram also illustrates the modulatory effects of natural compounds, including Resveratrol, Esculin, Citrus pectin, Magnolol, Baicalin, Ellagic acid, and Shikonin, in attenuating inflammation and renal injury

**Table 1:** Natural compounds targeting key signaling pathways involved in ischemic acute kidney injury (AKI)

Natural compound	Natural source	Primary targeted signaling pathway(s)	Downstream effects on inflammation / CAMs	Experimental AKI model	Delivery strategy	Translational readiness	Key references
Curcumin	*Curcuma longa*	TLR4/NF-κB; PI3K/Akt/mTOR	↓ TNF-α, IL-1β, IL-6; ↓ ICAM-1, VCAM-1; ↓ apoptosis	Renal I/R; cisplatin-AKI	Nanoparticles, micelles, liposomes	Preclinical (advanced)	Youn et al. 2006; Rogers et al. (2012) and Zhu et al. (2020)
Resveratrol	Grapes, berries	P2X7 receptor; NF-κB; NLRP3 inflammasome	↓ IL-1β, IL-18; ↓ inflammasome activation; ↓ CAM expression	Renal I/R; LPS-AKI	Polymeric nanoparticles	Preclinical	Nuka et al. (2018) and Nie et al. (2023)
Ellagic acid	Fruits, nuts	JAK1/2–STAT1/3	↓ STAT phosphorylation; ↓ cytokine amplification; ↓ apoptosis	Hypoxia-induced AKI	Conventional	Preclinical	Liu et al. (2020a)
Magnolol	*Magnolia officinalis*	JAK2/STAT3; oxidative stress pathways	↓ IL-6, TNF-α; ↓ tubular apoptosis	Renal I/R	Conventional	Preclinical	Tang et al. (2017) and Fu et al. (2021)
Baicalin	*Scutellaria radix*	P2X7–NLRP3 inflammasome	↓ IL-1β, IL-18; ↓ pyroptosis	Hyperuricemic & ischemic AKI	Conventional	Preclinical	Fu et al. (2024)
Dioscin	*Dioscorea* spp.	Hsp70 induction; TLR4/MyD88; COX-2	↓ ICAM-1; ↓ TNF-α, IL-6; ↓ leukocyte infiltration	Renal I/R	Conventional	Preclinical	Qi et al. (2015)
Hesperidin	Citrus fruits	TLR4/NF-κB/iNOS	↓ NF-κB p65; ↓ inflammatory cytokines; ↓ apoptosis	Renal I/R	Conventional	Preclinical	Meng et al. (2020)
Gentiopicroside	*Gentiana* spp.	TLR4 (MyD88/TRIF)	↓ TNF-α, IL-1β; ↓ CAM induction	LPS-induced AKI	Conventional	Preclinical	Shareef and Kathem (2022)
Oleic acid	*Camellia oleifera* seed oil	TLR4–MD2 complex	↓ TNF-α, IL-1β, IFN-γ; ↑ IL-10	LPS-AKI	Conventional	Preclinical	Zeng et al. (2020a)
Levistolide A	*Ligusticum chuanxiong*	TLR4/NF-κB	↓ IL-6, TNF-α; ↓ inflammatory infiltration	Glycerol-induced AKI	Conventional	Preclinical	Shi et al. (2024a)
Luteolin	Vegetables, herbs	NF-κB; MAPK	↓ TNF-α, IL-6; ↓ endothelial activation	Renal I/R	Conventional	Preclinical	Ozer Şehirli et al. (2009)
Ferulic acid	Grains, plants	NF-κB; oxidative stress pathways	↓ pro-inflammatory cytokines; ↓ CAM expression	Renal I/R	Conventional	Preclinical	Liu et al. (2017)
Pycnogenol	*Pinus pinaster* bark	NF-κB; oxidative stress	↓ TNF-α, IL-1β; ↓ endothelial dysfunction	Renal I/R	Conventional	Preclinical	Han et al. (2018)
Arctigenin	*Arctium lappa*	NF-κB; MAPK	↓ IL-6, TNF-α; ↓ leukocyte infiltration	Renal I/R	Conventional	Preclinical	Zhou et al. (2018)

Compound	Chemical class	Target of interaction	Evidence supporting use	Relative efficacy	Bioavailability	Toxicity	References
**Glycoside**							
Hydroxysafflor Yellow A	Chalcone Glycoside	TLR4, NF-κB, IL-1β, TNF-α	Suppresses Inflammation and apoptosis, improves renal function in ischemia–reperfusion model	$\downarrow$ BUN (67%) – $\downarrow$ Scr (71%)	Low	**Safe** however, showed a slight nephrotoxicit at 180 mg/kg after 90 days administration (Liu et al. 2004)	Bai et al. (2018) and Wang et al. (2022b)
Picroside II	Iridoid Glycoside	TLR4, NF-κB, TNF-α, IL-1β	Attenuates renal injury through blocking TLR4/NF-ÎºB pathway in ischemia–reperfusion model	$\downarrow$ BUN (75%) – $\downarrow$ Scr (58%)	Low	**Safe**	Wang et al. (2015)
Loganetin	Iridoid glycoside	TLR4, NF-κB, JNK/p38	Reduces inflammation and improves renal function in rhabdomyolysis-induced AKI	$\downarrow$ BUN (30–50%) – $\downarrow$ Scr (30–50%)	Low	**Safe**	Li et al. (2019b)
**Flavonoid**							
Xanthohumol	Flavonoid	Nrf2, NF-κB	Protects against AKI by activating Nrf2 and inhibiting NF-κB	$\downarrow$ BUN (30–70%) – $\downarrow$ Scr (30–70%)	Low	**Safe**	Li et al. (2018) and Ahmad et al. (2019)
Hesperidin	Flavonoid	Nrf2/ARE	Anti-inflammatory and anti-oxidative stress effects, significant protective role in AKI	$\downarrow$ BUN (75%) – $\downarrow$ Scr (33%)	Low	**Safe**	Chen et al. (2019)
Luteolin	Flavonoid	Nrf2	Activates Nrf2 and protects against renal injury	$\downarrow$ BUN (83%) – $\downarrow$ Scr (70%)	Low	**Safe at typical dietary levels – Genotoxic in high concentrations**	Tan et al. (2018)
Luteolin	Flavonoid	Bax, PUMA-α, caspase-3	Reduction in levels of apoptotic markers and increased p53 phosphorylation in cisplatin-AKI	$\downarrow$ BUN (75%) – $\downarrow$ Scr (30%)			Kang et al. (2011)
Galangin	Flavonoid	Nrf2/HO-1, NF-κB	Reduces oxidative stress and inflammation in cisplatin-induced AKI	$\downarrow$ BUN (73%) – $\downarrow$ Scr (35%)	Low	**Safe**	Huang et al. (2017)
Hyperin	Flavonoid	Nrf2, NF-κB	Reduces inflammatory and oxidative responses in CP-induced kidney injury	$\downarrow$ BUN (30–70%) – $\downarrow$ Scr (30–70%)	Low	**Safe**	Chao et al. (2016)
Quercetin	Flavonoid	NF-κB	Reduces inflammation in LPS-induced AKI	$\downarrow$ BUN (70%) – $\downarrow$ Scr (66%)	Low	**Safe**	Lu et al. (2021), Wang et al. (2020c)
Apigenin	Flavonoid	Bcl-2, p-Akt, P13K	Upregulated Bcl-2, p-Akt, P13K expression, and downregulated caspase-3 and Bax	$\downarrow$ BUN (52%) – $\downarrow$ Scr (50%)	Low	**Safe**	Wang et al. (2018a)
Tilianin	Flavonoid	ERK, BCL2L1	Suppressed ERK pathway activation and BCL2L1 expression in IRI-AKI	$\downarrow$ BUN (30–50%) – $\downarrow$ Scr (20–50%)	Moderate	**Safe**	Liu et al. (2022)
Genistein	Isoflavonoid	TLR4, TNF-α	Increased TLR4 and TNF-α expression levels in IRI-AKI	$\downarrow$ BUN (80%) – $\downarrow$ Scr (50%)	Low	**Safe**	Gholampour et al. (2020)
Anthocyanin	Flavonoid	IL-1β, IL-6, MCP-1, TNF-α	Decreased levels of inflammatory markers in IRI-AKI	$\downarrow$ BUN (69%) – $\downarrow$ Scr (66%)	Low	**Safe**	Li et al. (2021b)
Kaempferol	Flavonoid	TNF-α, iNOS, IL-12	Decreased inflammatory markers and phosphorylation of IBα in cisplatin-AKI	$\downarrow$ BUN (45%) – $\downarrow$ Scr (40%)	Low	**Safe**	Wang et al. (2020d)
**Phenolic compounds**							
Curcumin	Polyphenol	NF-κB	Attenuates inflammation and apoptosis in LPS-induced AKI mice	$\downarrow$ BUN (40%) – $\downarrow$ Scr (50%)	low	**Safe even at high doses (12 g/day) in humans and rodents**	Zhu et al. (2020) Anand et al. (2007)
Curcumin	Polyphenol	miR-181a	Down-regulates miR-181a expression, reduces renal tubular cell apoptosis				Huang et al. (2020)
Curcumin	Polyphenol	APPL1, Akt	Upregulation of APPL1 and inhibition of Akt phosphorylation in IRI-AKI				Fan et al. (2017)
Resveratrol	Polyphenol	Nrf2	Improves sepsis-induced AKI	$\downarrow$ BUN (57%)—$\downarrow$ Scr (57%)	Low (< 1%) (Walle 2011)	Low toxicity (Wang et al. 2018b)	Wang et al. (2017)
Resveratrol	Phenol	TLR4/ NF-κB	Suppresses inflammation and oxidative stress in IRI-induced AKI				Gao et al. (2018a)
Arbutin	Phenol	PI3K/Akt/Nrf2	Attenuates apoptosis and inflammation in LPS-induced AKI	$\downarrow$ BUN (43%)—$\downarrow$ Scr (37%)	High oral bioavailability (around 65%) in animal studies (Wang et al. 2024b)	• No remarkable toxicity, with an LD_50_ exceeding 2000 mg/kg (Mishra et al. 2025)	Zhang et al. (2021a)
Chlorogenic acid	Phenol Polyphenol	TLR4, NF-κB, MyD88	Inhibits TLR4/NF-κB signaling, and reduces secretion of inflammatory cytokines in LPS-induced AKI model	$\downarrow$ BUN (52%—64%)—$\downarrow$ Scr (36%—64%)	• low direct bioavailability due to its chemical structure, largely depending on metabolism by the gut flora (Gonthier et al. 2003)	• Safe at doses within the normal range of dietary exposure (Behne et al. 2023)	Ye et al. (2017)
Salvianolic acid A	Polyphenol	TLR4 (Toll-Like Receptor 4), MyD88, NF-κB, JNK1/2	Ameliorates inflammation, prevents glomerular atrophy, reduces oxidative stress, and inhibits macrophage infiltration in AKI	BUN (60%—76%)—$\downarrow$ Scr (56%—83%) (Wu et al. 2023)	Low	• Primarily affects the liver and kidneys, leading to liver cell necrosis and kidney damage in animal studies (Yang et al. 2022)	Zeng et al. (2020b)
**Saponin**							
Dioscin	Steroidal saponin	miR-34a	Reverses the up-regulation of miR-34a induced by cisplatin, increases antioxidation activities	BUN (40% -70%)—$\downarrow$ Scr (38%- 59%)	Very low (Salunkhe et al. 2021)	• No-observed-adverse-effect level (NOAEL) and the lowest-observed-adverse-effect level (LOAEL) of dioscin are estimated to be 300 mg/kg/day for female and male rats, respectively (Xu et al. 2012)	Zhang et al. (2017a, b, c)

**Table 2:** Nanomaterial-based immunomodulatory treatments in managing AKI (Zhang et al. 2025a, b)

Biologically derived, biodegradable, and immunomodulatory materials	Non-biodegradable, immunoactive nanomaterials	Biodegradable, immunologically inert nanoparticles	Non-functionalized or specifically modified nanoparticles
e.g. cell membrane–derived nanoparticles, extracellular vesicles, and exosomes	e.g. Metal nanoparticles (Au, Ag, Cu, FeO₂) and functionalized carbon nanotubes	e.g. polymers, and dendrimers	e.g. TPP-modified ceria NPs, ROS-responsive polymer-coated atorvastatin-encapsulated ceria NPs (Atv/PTP-TCeria), and Se@BSA NPs

Despite increasing interest, the clinical translation of natural compounds for AKI remains limited by several challenges, including poor bioavailability, lack of standardized formulations, insufficient toxicity profiling, and incomplete evaluation of drug–natural product interactions (Anand et al. 2007; Bertoncini-Silva et al. 2024). Furthermore, many existing reviews focus on individual pathways or specific compound classes, providing fragmented insight into the integrated molecular landscape of AKI.

Therefore, this review aims to provide a comprehensive and mechanistically integrated analysis of natural compounds targeting ischemic AKI. By systematically linking distinct classes of natural products to their molecular targets and signaling pathways, this work seeks to clarify their therapeutic potential, translational limitations, and future research directions. In doing so, we propose a structured framework to support the rational development of natural compound–based interventions for AKI.

### Ischemic AKI

AKI is a clinical syndrome characterized by a rapid decline in renal function occurring over hours to weeks, and in some definitions up to less than three months. It is clinically diagnosed based on an abrupt reduction in glomerular filtration rate (GFR), an acute rise in serum creatinine (SCr), and/or a decrease in urine output (Thomas et al. 2015). AKI is a frequent complication in hospitalized patients, affecting approximately 5–7.5% of general admissions and up to 50–60% of critically ill individuals in intensive care units (Rodrigues et al. 2013; Hoste et al. 2015; Gameiro et al. 2020) (Fig. 1).

Despite advances in supportive care, AKI remains associated with unacceptably high mortality rates. In intensive care settings, mortality can exceed 50%, and survivors face a markedly increased risk of developing chronic kidney disease (CKD) and end-stage renal disease (ESRD) (Coca et al. 2009; Hsu et al. 2009; Ishani et al. 2009). Global epidemiological analyses estimate unadjusted mortality rates of approximately 23.9% in adults and 13.8% in pediatric populations, with mortality increasing proportionally with AKI severity (Uchino et al. 2005; Susantitaphong et al. 2013). In the United States alone, AKI accounts for nearly 600,000 hospital admissions annually, while worldwide it contributes to almost two million deaths each year (Gammelager et al. 2012; Masewu et al. 2016).

Renal I/R injury represents one of the most prevalent and clinically significant causes of AKI. Ischemia arises from a sudden reduction in renal blood flow, leading to inadequate oxygen and nutrient delivery and impaired removal of metabolic waste products. Subsequent reperfusion, although essential for tissue survival, paradoxically exacerbates renal injury through oxidative stress, inflammatory activation, and microvascular dysfunction (Mehta et al. 2004; Devarajan 2006; Haddad et al. 2019). Histopathologically, I/R-induced AKI is characterized by tubular epithelial cell injury, loss of brush borders, tubular dilation, epithelial detachment, and interstitial edema, while glomerular architecture is often relatively preserved (Castaneda et al. 2003; Safirstein 2004; Hong et al. 2017).

Cell death in ischemic AKI occurs through both necrotic and apoptotic mechanisms. Necrosis predominantly affects the outer medulla, whereas apoptosis has been documented in both proximal and distal tubular segments (Castaneda et al. 2003; Safirstein 2004). In parallel, vascular congestion, endothelial injury, and leukocyte adhesion develop within peritubular capillaries, further impairing renal perfusion and amplifying inflammatory injury (Friedewald and Rabb2004; Qu and Jiao 2023). Collectively, these events establish a self-perpetuating cycle of hypoxia, inflammation, and cellular damage that drives AKI progression.

Among the multiple pathological processes underlying ischemic AKI, disruption of the renal microcirculation has emerged as a central determinant of injury severity and functional recovery. The unique vascular architecture of the kidney renders specific regions particularly susceptible to ischemia, making microvascular dysfunction a critical contributor to both acute damage and maladaptive repair.

### Renal microcirculation injury in ischemic AKI

The renal microcirculation plays a central role in the pathogenesis of ischemic AKI and is a critical determinant of both injury severity and recovery. Owing to the kidney’s unique vascular architecture, regional alterations in blood flow are more pathophysiologically relevant than changes in total renal perfusion (Bonventre and Weinberg 2003; Schrier and Wang 2004). The outer medulla is particularly vulnerable to ischemic injury because it receives only 5–10% of total renal blood flow despite having high metabolic demands associated with active tubular transport (Le Dorze et al. 2009; Kwiatkowska et al. 2023).

During ischemia, oxygen deprivation leads to ATP depletion and impaired Na⁺/K⁺-ATPase activity within tubular epithelial cells, particularly in the medullary thick ascending limb. This adaptive downregulation reduces sodium reabsorption and oxygen consumption but simultaneously disrupts tubular transport function. The resulting increase in distal sodium delivery activates tubuloglomerular feedback, inducing afferent arteriolar vasoconstriction and further reducing glomerular filtration rate (Briggs et al. 1990; Carlström et al. 2015). Although initially protective, this response contributes to sustained hypoperfusion and exacerbates medullary hypoxia.

Following reperfusion, restoration of microvascular blood flow remains incomplete and heterogeneous. Endothelial swelling, pericyte contraction, erythrocyte aggregation, and capillary obstruction collectively impair peritubular capillary perfusion, a phenomenon often referred to as the “no-reflow” effect (Hellberg et al. 1990; Olof et al. 1991; Evans et al. 2013). Experimental studies have demonstrated that medullary capillary density is among the earliest microvascular features affected after ischemic injury, with persistent rarefaction contributing to chronic hypoxia, fibrosis, and progression to CKD (Zhang et al. 2008; Basile et al. 2008; Kwiatkowska et al. 2023).

Endothelial dysfunction is a hallmark of microcirculatory injury in ischemic AKI. I/R reduces nitric oxide bioavailability while increasing vasoconstrictors such as endothelin-1, angiotensin II, and thromboxane A₂, leading to sustained vasoconstriction and impaired autoregulation of renal blood flow (Brooks 1996; Kwon et al. 2009; da Silveira et al. 2010). Inflammatory activation of endothelial cells further promotes leukocyte adhesion, increased vascular permeability, and interstitial edema, which mechanically compress capillaries and aggravate hypoxia (Bonventre and Zuk 2004; Rabelink et al. 2010).

Importantly, microvascular injury is not merely a consequence of ischemic AKI but a key driver of maladaptive repair. Loss of peritubular capillaries, downregulation of vascular endothelial growth factor (VEGF), and upregulation of angiogenesis inhibitors collectively impair vascular regeneration and favor fibrotic remodeling (Basile 2007; Basile et al. 2008). These microcirculatory alterations establish a mechanistic link between acute ischemic injury and long-term renal dysfunction, underscoring the importance of therapeutic strategies that preserve or restore renal microvascular integrity.

Beyond its hemodynamic consequences, renal microcirculatory dysfunction serves as a critical initiating event that links I/R injury to the downstream molecular and cellular mechanisms of ischemic AKI. Persistent hypoxia, endothelial activation, and capillary rarefaction create a pro-inflammatory microenvironment that promotes tubular epithelial cell stress and death, leading to the release of damage-associated molecular patterns (DAMPs). These endogenous danger signals activate innate immune pathways in resident renal cells and infiltrating leukocytes, thereby amplifying inflammatory cascades and perpetuating tissue injury (Bonventre and Zuk 2004; Akcay et al. 2009).

Consequently, ischemic AKI evolves from a primary microvascular insult into a complex inflammatory disorder characterized by cytokine overproduction, leukocyte recruitment, endothelial–epithelial crosstalk, and activation of multiple intracellular signaling pathways. Understanding how these processes are orchestrated at the molecular level is essential for identifying therapeutic targets that can interrupt the progression of acute injury toward maladaptive repair and chronic kidney disease. The following section, therefore, focuses on the key pathogenic mechanisms underlying ischemic AKI, with particular emphasis on inflammatory signaling networks and their regulatory pathways.

### Pathogenesis of ischemic AKI

AKI is no longer regarded as a purely hemodynamic disorder but rather as a complex, tightly regulated biological process driven by the interplay between microvascular dysfunction, tubular epithelial injury, and dysregulated immune signaling. Following I/R, renal injury evolves through a coordinated sequence of molecular events that activate innate immune responses, amplify inflammatory signaling, and ultimately determine the balance between adaptive repair and maladaptive fibrosis (Bonventre and Zuk 2004; Bonventre and Yang 2011).

At the core of this process lies signal transduction, whereby cellular stress and tissue damage are translated into inflammatory and apoptotic responses. These signaling pathways are initiated by ischemia-induced hypoxia and oxidative stress and are propagated through pattern recognition receptors, cytokine networks, and intracellular kinase cascades.

#### Endothelial and tubular stress as the initiating signaling event

Renal ischemia results in ATP depletion, mitochondrial dysfunction, and cytoskeletal disruption in tubular epithelial cells, particularly within the metabolically vulnerable outer medulla. Concurrently, endothelial cells exposed to hypoxia and shear stress imbalance undergo activation and dysfunction, characterized by reduced nitric oxide bioavailability and increased production of vasoconstrictors such as endothelin-1, angiotensin II, and thromboxane A₂ (Brooks 1996; Kwon et al. 2009; da Silveira et al. 2010).

These early cellular stress responses initiate intracellular signaling cascades that sensitize both tubular and endothelial cells to inflammatory injury. Importantly, endothelial dysfunction amplifies ischemic damage by promoting leukocyte adhesion, increasing vascular permeability, and exacerbating interstitial edema, thereby reinforcing hypoxia and cellular stress (Rabelink et al. 2010).

#### DAMP-driven innate immune activation and TLR signaling

Tubular cell necrosis and apoptosis following I/R injury lead to the release of damage-associated molecular patterns (DAMPs), including HMGB1, mitochondrial DNA, and extracellular ATP. These endogenous danger signals activate pattern recognition receptors, particularly Toll-like receptors (TLRs), expressed on tubular epithelial cells, endothelial cells, resident macrophages, and infiltrating leukocytes (Wu et al. 2007; Kurts et al. 2013; Mulay et al. 2016).

Among the TLR family, TLR4 plays a central role in ischemic AKI by activating both MyD88-dependent and TRIF-dependent signaling pathways. Activation of TLR4 leads to phosphorylation of MAPKs and IκB kinase (IKK), culminating in nuclear translocation of NF-κB and AP-1 transcription factors. This signaling cascade induces robust transcription of pro-inflammatory mediators, including tumor necrosis factor-α (TNF-α), interleukin (IL)-1β, and IL-6 (Akira and Takeda 2004; Jang and Rabb 2009; Andrade-Oliveira et al. 2012).

#### Cytokine amplification and JAK/STAT crosstalk

The cytokine milieu generated downstream of TLR activation serves not only as an effector mechanism but also as a secondary signaling amplifier. IL-6, in particular, exerts pleiotropic effects in ischemic AKI through activation of the JAK/STAT pathway. Binding of IL-6 to its receptor complex activates gp130-associated JAK kinases, leading to STAT3 phosphorylation and nuclear translocation (Peters et al. 1998).

STAT3 signaling contributes to tubular cell survival, inflammatory gene expression, and immune cell recruitment, highlighting its dual role in injury and repair (Akcay et al. 2009; Correa-Costa et al. 2012). Dysregulated or sustained activation of the JAK/STAT axis has been implicated in prolonged inflammation and progression toward chronic kidney disease (CKD), underscoring its importance as a therapeutic target.

#### Integration of oxidative stress, NF-κB, and cell death pathways

Reactive oxygen species (ROS) generated during reperfusion further intensify inflammatory signaling by activating redox-sensitive transcription factors, including NF-κB. NF-κB signaling regulates the expression of numerous downstream effectors, including cyclooxygenase-2 (COX-2), inducible nitric oxide synthase (iNOS), and adhesion molecules, thereby linking oxidative stress to inflammation and leukocyte recruitment (Cook et al. 2004; Funk 2001).

In parallel, mitochondrial injury and sustained oxidative stress shift the balance toward apoptotic and necrotic cell death by modulating Bcl-2 family proteins and activating caspases. The convergence of inflammatory signaling and cell death pathways reinforces tubular damage and perpetuates the inflammatory microenvironment characteristic of ischemic AKI.

Collectively, these interconnected signaling networks transform ischemic AKI from an acute metabolic insult into a self-amplifying inflammatory disorder. However, the pathogenic effects of these molecular pathways are ultimately exerted at the cellular interface, where activated endothelial cells, leukocytes, and injured tubular epithelial cells interact within the renal microvasculature. Central to this process is the regulated expression of cell adhesion molecules (CAMs), which govern leukocyte recruitment, endothelial–epithelial crosstalk, and spatial localization of inflammation within the injured kidney.

Accordingly, a detailed understanding of CAM-mediated interactions provides critical insight into how intracellular signaling pathways translate into tissue-level injury and dysfunction. The following section, therefore, focuses on the role of cell adhesion molecules in ischemic AKI, highlighting their mechanistic contributions to inflammatory amplification and their potential as therapeutic targets.

### Cell adhesion molecules as downstream effectors of inflammatory signaling in ischemic AKI

Following I/R injury, activation of intracellular inflammatory signaling pathways is translated into tissue-level injury through tightly regulated interactions between endothelial cells, leukocytes, and tubular epithelial cells. Cell adhesion molecules (CAMs) serve as the critical molecular interface through which inflammatory signals orchestrate leukocyte recruitment, endothelial activation, and the spatial propagation of renal injury. Thus, CAMs function not merely as passive markers of inflammation but as active downstream effectors of signaling cascades initiated during ischemic AKI (Bonventre and Yang 2011; Soares et al. 2019).

CAMs are broadly classified into four major families: selectins, integrins, members of the immunoglobulin superfamily (including ICAM-1 and VCAM-1), and cadherins. Each class plays a distinct yet coordinated role in mediating leukocyte–endothelial interactions and epithelial integrity within the injured kidney (Alberts et al. 2002; Singh et al. 2021).

#### Signaling-dependent induction of CAM expression

Pro-inflammatory signaling pathways activated during ischemic AKI, particularly TLR/NF-κB, JAK/STAT, and MAPK cascades, directly regulate CAM gene transcription. NF-κB activation induces the expression of ICAM-1, VCAM-1, E-selectin, and P-selectin on renal endothelial cells, thereby establishing a pro-adhesive vascular phenotype that facilitates leukocyte tethering and firm adhesion (Rabb et al. 1997; Cook et al. 2004).

In parallel, cytokines such as TNF-α and IL-1β amplify CAM expression through autocrine and paracrine signaling, reinforcing endothelial activation and sustaining inflammatory cell recruitment (Akcay et al. 2009). This signaling-driven upregulation of CAMs provides a mechanistic link between intracellular inflammatory pathways and the spatial localization of renal injury.

#### Selectins and the initiation of leukocyte recruitment

Selectins mediate the earliest steps of leukocyte recruitment by facilitating transient, low-affinity interactions between circulating leukocytes and activated endothelial cells. In ischemic AKI, endothelial P-selectin and E-selectin are rapidly upregulated, promoting leukocyte rolling along the peritubular capillary wall (Burne-Taney and Rabb 2003; Siddiqui et al. 2023).

Experimental models have demonstrated that inhibition of P-selectin significantly attenuates leukocyte infiltration, reduces tubular injury, and improves renal function following I/R (Singbartl et al. 2000; Hayashi et al. 2001). These findings underscore the importance of selectins as early, signaling-regulated mediators of inflammatory cell recruitment in ischemic AKI.

#### Integrins and firm adhesion: stabilizing inflammatory cell infiltration

Following selectin-mediated rolling, leukocyte integrins undergo conformational activation in response to chemokine signaling, enabling firm adhesion to endothelial CAMs. Integrins such as αLβ2 (LFA-1) and αMβ2 (Mac-1) bind to ICAM-1 and VCAM-1, stabilizing leukocyte attachment and facilitating transendothelial migration (Danen 2013; Pang et al. 2023).

In ischemic AKI, integrin-mediated adhesion not only enhances leukocyte infiltration but also contributes to microvascular congestion and impaired capillary perfusion. Pharmacological or genetic inhibition of integrin signaling has been shown to reduce I/R injury, limit fibrosis, and preserve renal function, highlighting integrins as key effectors of inflammatory damage (Yago et al. 2015; Basta et al. 2020).

#### Immunoglobulin superfamily CAMs: ICAM-1 and VCAM-1 as central amplifiers

Among CAMs, ICAM-1 and VCAM-1 play a central role in amplifying inflammatory injury in ischemic AKI. ICAM-1 is minimally expressed under physiological conditions but is markedly upregulated in response to ischemia and inflammatory signaling. Its interaction with leukocyte integrins promotes firm adhesion, transmigration, and sustained inflammatory activation (Soares et al. 2019; Bui et al. 2020).

Clinical and experimental studies have shown that elevated circulating levels of soluble ICAM-1 and VCAM-1 correlate with ischemic injury severity and adverse outcomes, supporting their utility as biomarkers of endothelial activation and inflammation (Danton and Dietrich 2003; Lehmann et al. 2022). Importantly, ICAM-1 blockade has been associated with reduced leukocyte infiltration and attenuation of renal injury in experimental models.

#### Cadherins and epithelial integrity in ischemic AKI

In contrast to CAMs that promote inflammation, cadherins, particularly E-cadherin and N-cadherin, are essential for maintaining epithelial integrity. Ischemic injury disrupts cadherin-mediated cell–cell adhesion in renal tubules, contributing to epithelial detachment, loss of polarity, and impaired repair (Perez and Nelson 2004; Nürnberger et al. 2010).

Restoration of cadherin expression has been shown to mitigate tubular injury and suppress apoptosis in experimental AKI models, highlighting the dual role of CAMs in both injury propagation and tissue repair (Gao et al. 2018b; Ni et al. 2019).

#### CAMs as therapeutic and translational targets in ischemic AKI

Collectively, CAMs represent a critical convergence point where upstream inflammatory signaling pathways are translated into cellular interactions and tissue injury. By governing leukocyte recruitment, endothelial–epithelial crosstalk, and microvascular integrity, CAMs occupy a strategic position in the pathogenesis of ischemic AKI.

This central role renders CAMs attractive therapeutic targets. Modulation of CAM expression or function, either directly or indirectly through upstream signaling pathways, offers the potential to attenuate inflammation, preserve microcirculation, and promote renal recovery. Importantly, many natural compounds discussed in subsequent sections exert their renoprotective effects by suppressing CAM expression by inhibiting NF-κB, JAK/STAT, and related signaling cascades.

Given their pivotal role as downstream effectors of inflammatory signaling, CAMs provide a mechanistic framework for understanding how pharmacological interventions can disrupt leukocyte–endothelial interactions and attenuate ischemic renal injury. Notably, a growing body of evidence indicates that several natural compounds exert potent renoprotective effects by targeting CAM expression and the upstream signaling pathways that regulate them. The following section, therefore, examines the impact of natural products on inflammatory signaling and CAM-mediated processes in ischemic AKI.

##### Natural compounds targeting inflammatory signaling and CAM-mediated injury in ischemic AKI (Fig. 2)

Given the central roles of inflammatory signaling and CAM-mediated leukocyte recruitment in the pathogenesis of ischemic AKI, therapeutic strategies that modulate these pathways hold significant promise. Natural compounds have attracted growing attention in this context because of their pleiotropic biological activities, low systemic toxicity, and ability to target multiple components of the inflammatory cascade simultaneously. Importantly, many natural products exert renoprotective effects by interfering with upstream inflammatory signaling pathways that regulate CAM expression and leukocyte-endothelial interactions (Li et al. 2019b; Kang et al. 2021).

#### Natural modulators of TLR/NF-κB signaling 

Activation of the TLR/NF-κB axis represents a pivotal initiating event in ischemic AKI–associated inflammation. Several natural compounds have been shown to attenuate renal injury by suppressing TLR activation or downstream NF-κB signaling, thereby reducing pro-inflammatory cytokine production and CAM expression. Beyond canonical inflammatory activation, recent evidence indicates that TLR4/NF-κB signaling in ischemic AKI is tightly coupled to mitochondrial dysfunction, amplification of oxidative stress, and regulated cell death pathways, including ferroptosis and pyroptosis. This positions the TLR/NF-κB axis as a central integrative hub linking innate immune sensing to downstream metabolic and death programs in injured renal tubular and endothelial cells (Chen et al. 2025; Tian et al. 2025).

Levistolide A, an active constituent of *Ligusticum chuanxiong*, significantly ameliorated glycerol-induced AKI in mice by inhibiting TLR4/NF-κB signaling. This effect was associated with reduced renal expression of TLR4, IL-6, and TNF-α, leading to attenuation of inflammatory infiltration and tubular injury (Shi et al. 2024a).

Oleic acid is the major monounsaturated fatty acid in *Camellia oleifera* seed (camellia) oil, and oleic acid-rich camellia oil has been reported to attenuate LPS-induced AKI by downregulating TLR4-linked inflammatory signaling (including NF-κB/AP-1/IRF3) and suppressing NLRP3 inflammasome activation, accompanied by reduced pro-inflammatory cytokine responses and improved renal injury indices (Zeng et al. 2020a). Consistently, pharmacological oleic acid administration has also been shown to mitigate LPS-induced AKI by restraining inflammation and oxidative stress through Ras/MAPKs/PPAR-γ-associated mechanisms, supporting a context-dependent anti-inflammatory action of oleic acid-related interventions in endotoxemic AKI (Zhang et al. 2022). Importantly, oleic acid exhibits model- and exposure-dependent bidirectionality: intravenous oleic acid is widely used as a lipotoxic trigger in experimental acute organ injury and has been reported to induce lung and kidney injury, whereas certain interventions (e.g., AT1R blockade) may partially mitigate oleic acid-induced tissue damage in specific settings (Talebi et al. 2021). Moreover, oleic acid-driven lipotoxicity in renal tubular cells has been mechanistically linked to mitochondrial ROS generation and the stress adaptor p66Shc pathway, highlighting that “oleic acid effects” cannot be generalized without specifying dose, formulation, and disease context (Arany et al. 2013). Recent rat studies using oleic acid to induce AKI further report that hispidulin reverses oleic acid-associated oxidative stress, inflammatory gene expression (e.g., IL-6, NF-κB), and tubular injury markers (e.g., KIM-1), again underscoring oleic acid as a lipotoxic insult model in vivo (Avci et al. 2025a, b). These distinctions are highly relevant to ischemic and necroinflammatory AKI, where DAMP-driven innate immune activation (including TLR4-associated pathways) can amplify cytokine release and tissue injury, creating self-propagating inflammatory loops (Mulay et al. 2016). Accordingly, contemporary reviews of AKI therapeutics emphasize that natural compounds capable of disrupting TLR4-centered signaling nodes (at the receptor complex level or downstream adaptor/effector steps) may be particularly valuable for interrupting necroinflammatory amplification in AKI (Chen et al. 2025). In parallel, electrophilic oleic acid derivatives such as nitro-oleic acid (oleic acid-NO₂) have shown renoprotective actions in tubular and glomerular injury models via Nrf2-driven antioxidant programs and suppression of NADPH oxidase activity, illustrating that oleic acid-derived lipid mediators can engage cytoprotective signaling networks distinct from those activated by lipotoxic oleic acid overload (Nie et al. 2016; Liu et al. 2013).

Curcumin, a polyphenolic compound derived from Curcuma longa, represents one of the most extensively studied natural modulators of inflammatory signaling in AKI. Multiple experimental studies have demonstrated that curcumin attenuates ischemic and toxic renal injury by suppressing TLR4/NF-κB activation, reducing oxidative stress, and downregulating pro-inflammatory cytokines, including IL-1β, IL-6, and TNF-α (Youn et al. 2006; Rogers et al. 2012; Avila-Rojas et al. 2020; Zhu et al. 2020; Hui et al. 2021). In addition, curcumin has been shown to reduce COX-2 and iNOS expression, thereby indirectly limiting CAM induction and leukocyte recruitment (Zhao and Shen 2021). Notably, curcumin-mediated inhibition of TLR4/NF-κB signaling has also been linked to restoration of mitochondrial redox balance and activation of Nrf2-dependent antioxidant responses, suggesting coordinated regulation of inflammatory and cytoprotective pathways. This dual modulation may be particularly advantageous in ischemic AKI, where oxidative stress and inflammation act synergistically to exacerbate tubular injury (Fan et al. 2017; Tian et al. 2025).

Hyperoside (also referred to as hyperin; quercetin-3-O-galactoside), a naturally occurring flavonol glycoside, has demonstrated robust renoprotective effects across multiple experimental models of acute kidney injury (AKI). In endotoxemic and cisplatin-induced AKI, hyperoside/hyperin consistently suppressed TLR4-dependent inflammatory signaling, inhibited NF-κB activation, and attenuated NLRP3 inflammasome engagement, resulting in reduced renal production of pro-inflammatory cytokines such as TNF-α, IL-1β, and IL-6 (Gong et al. 2016; Chao et al. 2016). Beyond inflammation control, hyperoside alleviated oxidative stress and tubular cell death by activating the Nrf2/HO-1 antioxidant axis in cisplatin-induced AKI and by preserving mitochondrial integrity through inhibition of OMA1-mediated OPA1 cleavage and pathological mitochondrial fission in ischemia–reperfusion injury (Chao et al. 2016; Wu et al. 2019). Notably, in cisplatin nephrotoxicity, hyperoside additionally enhanced renal organic anion transporter-1 (Oat1) expression and function by upregulating HNF-1α and PXR, thereby promoting the urinary excretion of uremic toxins such as indoxyl sulfate and directly mitigating the tubular toxic burden (Yuan et al. 2023). Collectively, these findings position hyperoside as a multi-target renoprotective agent that integrates anti-inflammatory, antioxidant, mitochondrial-stabilizing, and transporter-modulating mechanisms in AKI.

Baicalein and its glycoside baicalin, flavonoids derived from Scutellaria baicalensis, have been widely demonstrated to exert renoprotective effects across ischemic, toxic, and inflammatory models of acute kidney injury (AKI) by attenuating innate immune–driven inflammatory signaling and oxidative stress (Wu et al. 2015; Sahu et al. 2015; Chen et al. 2018). Mechanistically, baicalein-based interventions suppress TLR4/MyD88-dependent NF-κB activation, resulting in reduced renal production of pro-inflammatory cytokines, including TNF-α, IL-1β, and IL-6, as well as downregulation of endothelial and tubular adhesion molecules, thereby limiting leukocyte recruitment and microvascular inflammation in injured kidneys (Sahu et al. 2015; Wu et al. 2015; Elmarakby et al. 2019). In addition, emerging evidence indicates that baicalein and baicalin modulate purinergic signaling–driven inflammatory amplification, particularly by inhibiting the Panx1/P2X7 receptor axis, thereby suppressing inflammasome activation and pyroptotic cell death in renal tubular epithelial cells (Fu et al. 2024). More recent studies further demonstrate that baicalein attenuates lipid peroxidation–driven ferroptosis, including ALOX12-dependent pathways, and restores mitochondrial redox homeostasis, positioning baicalein as a multifunctional regulator at the intersection of innate immune sensing, oxidative stress, and regulated cell death pathways in AKI (Guo et al. 2024a, b; Liang et al. 2024; Zhou et al. 2025).

Paeoniflorin, a monoterpene glycoside isolated from Paeonia lactiflora, represents a well-characterized example of TLR4/NF-κB–targeted renoprotection in experimental acute kidney injury (AKI). In ischemia–reperfusion and inflammatory AKI models, paeoniflorin treatment suppressed renal TLR4 expression, inhibited NF-κB activation, and significantly reduced pro-inflammatory cytokine production, including tumor necrosis factor-α (TNF-α) and interleukin-1β (IL-1β), thereby attenuating inflammatory cell infiltration and microvascular dysfunction in injured kidneys (Zhang et al. 2018b; Wang et al. 2020b). Consistent with these findings, paeoniflorin has also been shown to mitigate oxidative stress, regulate cell death pathways (including ferroptosis and necroptosis), and macrophage-driven renal inflammation, further supporting its role as a multifunctional modulator of innate immune–mediated renal injury (Ma et al. 2023; Zhu et al. 2025; Cao et al. 2023).

Quercetin, one of the most abundant dietary flavonoids, has been consistently reported to exert renoprotective effects in diverse experimental models of acute kidney injury (AKI) by attenuating oxidative stress– and innate immune–driven inflammatory signaling in renal endothelial and tubular cells (Bagheri et al. 2023; Zeng et al. 2023; Albrakati 2025). Mechanistically, quercetin treatment suppresses TLR4-dependent NF-κB activation, leading to a marked reduction in pro-inflammatory cytokine production and downregulation of endothelial adhesion molecules, including intercellular adhesion molecule-1 (ICAM-1) and vascular cell adhesion molecule-1 (VCAM-1), thereby limiting neutrophil infiltration and inflammatory cell recruitment in injured kidneys (Alhusaini et al. 2019; Jin et al. 2024; Lu et al. 2021). In parallel, quercetin has been shown to preserve renal microvascular and tubular integrity by modulating SIRT1/NF-κB–associated transcriptional programs and by inhibiting regulated cell death pathways, including ferroptosis and cuproptosis, further supporting a direct mechanistic link between TLR4/NF-κB suppression, CAM regulation, and microcirculatory protection in experimental AKI (Wang et al. 2020c; Shi et al. 2024a, b; Elsherbiny et al. 2025).

**Salidroside**, a phenylpropanoid glycoside isolated from Rhodiola rosea, has emerged as a multifunctional renoprotective agent in experimental acute kidney injury (AKI). In ischemia–reperfusion and hypoxia/reoxygenation models, salidroside consistently suppressed TLR4/NF-κB signaling, leading to reduced transcription of pro-inflammatory cytokines, attenuation of oxidative stress, and inhibition of tubular epithelial apoptosis (Sun et al. 2018; Fan et al. 2022; Pan et al. 2023). In parallel, salidroside has been shown to modulate endothelial–immune interactions and microvascular inflammation, as evidenced by inhibition of TLR4/NF-κB– and MAPK-dependent inflammatory programs and improvement of renal microenvironmental injury, processes closely linked to adhesion molecule–mediated leukocyte recruitment (Li et al. 2019d). More recent studies further extend its protective profile by demonstrating that salidroside limits regulated cell death pathways, including ferroptosis, pyroptosis, and PANoptosis, through coordinated regulation of PI3K/AKT, Nrf2-dependent antioxidant signaling, and hypoxia-responsive pathways, thereby preserving tubular and microvascular integrity in ischemic, septic, and crystal-associated AKI models (Tang et al. 2023; Zhen et al. 2026; Zhan et al. 2025).

**Fig. 4:**
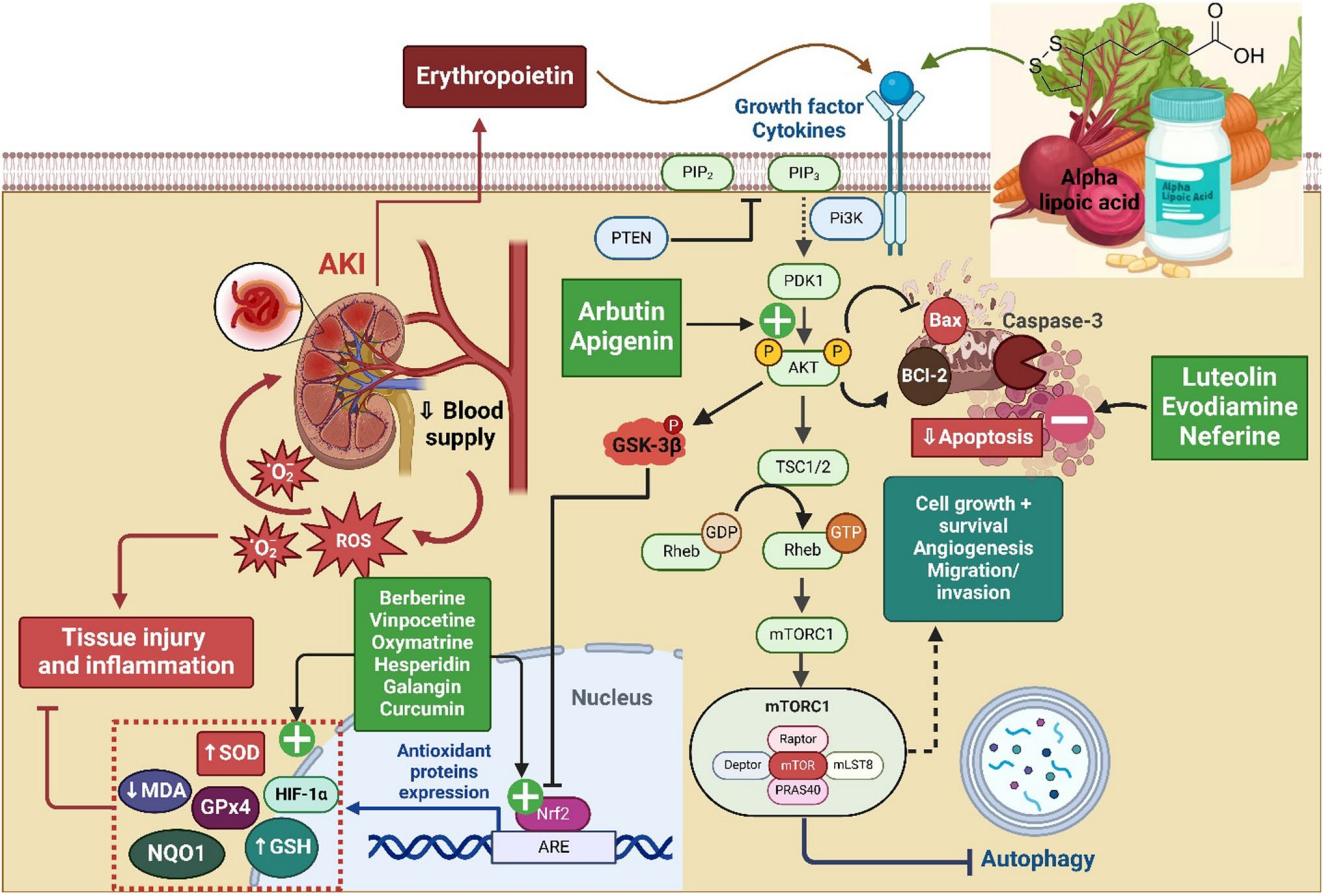
Illustrates the roles of PI3K/AKT/mTOR signaling, and Nrf2-mediated antioxidant defense and apoptosis regulation, as well as the protective effects of natural compounds in modulating these pathways to mitigate renal injury

**Fig. 5:**
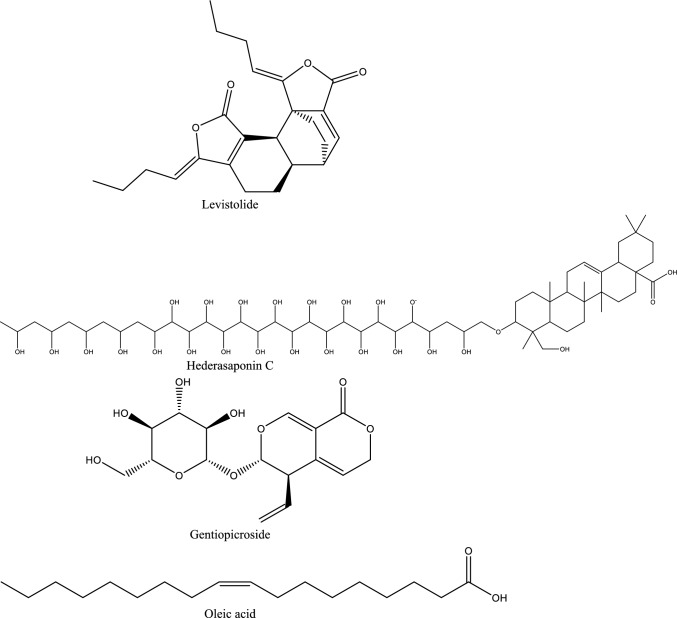

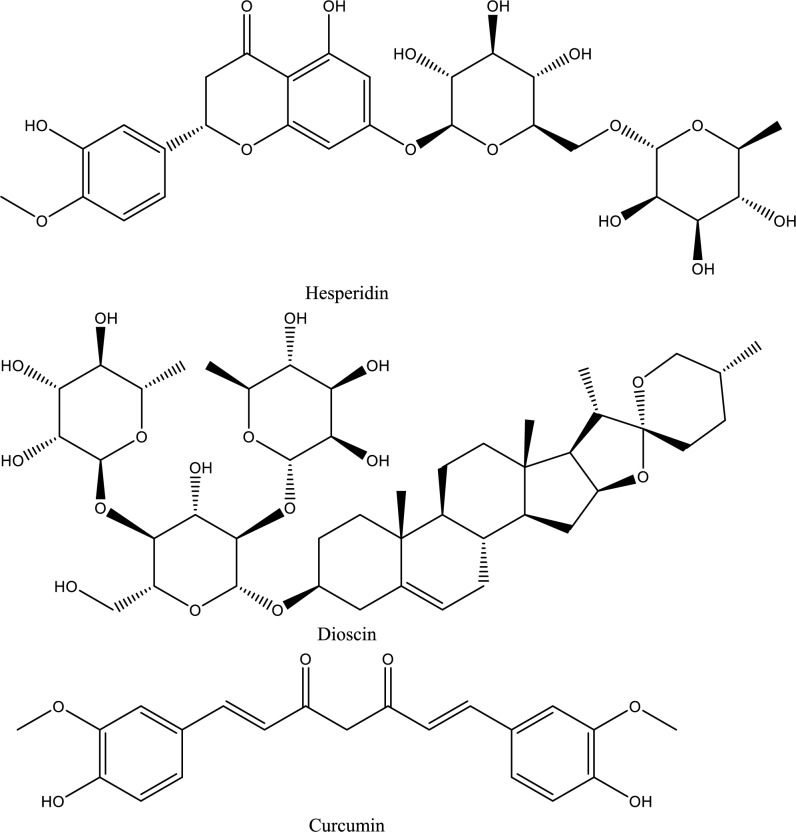

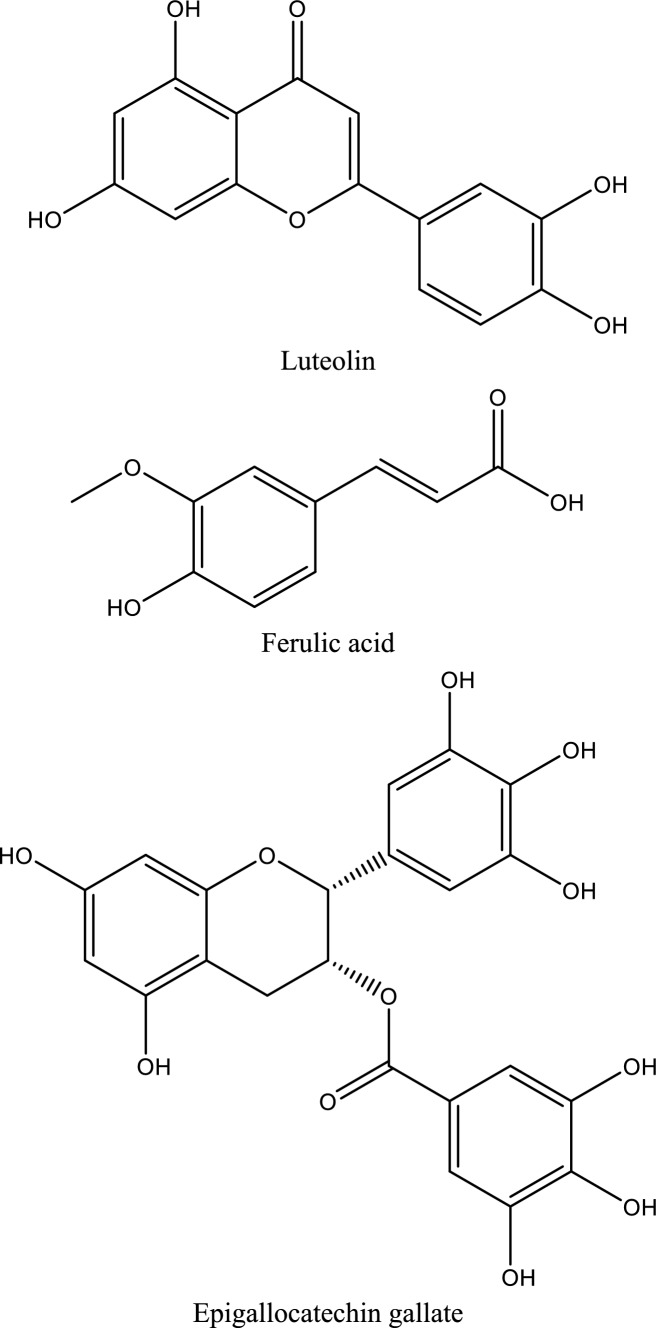

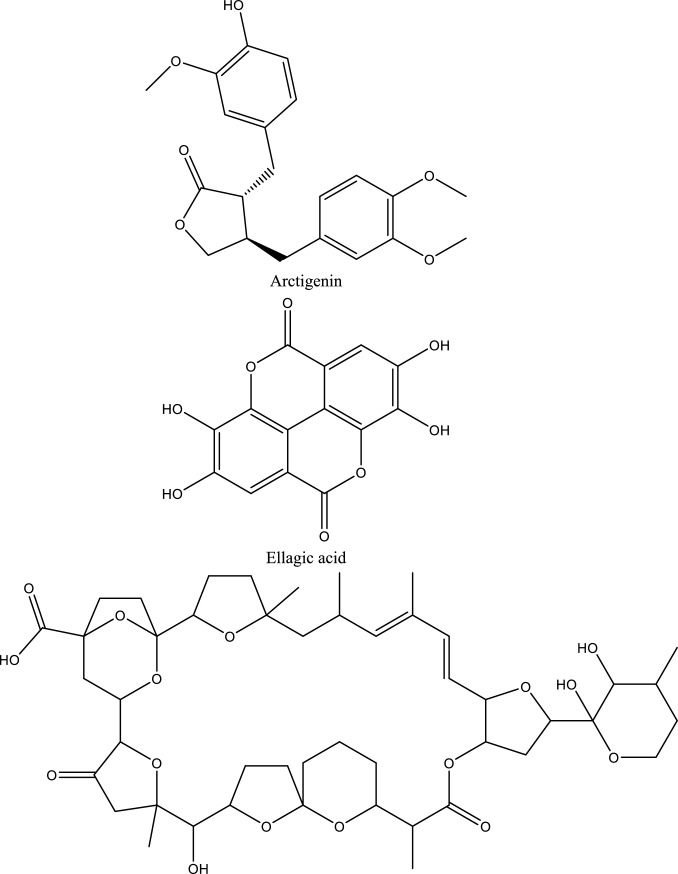

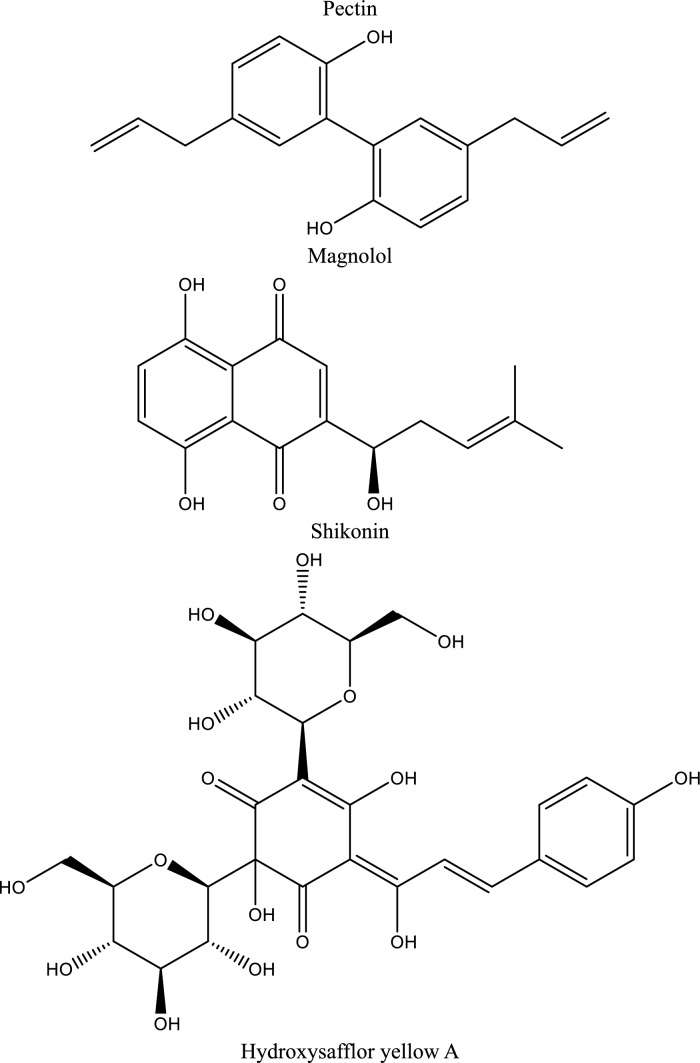

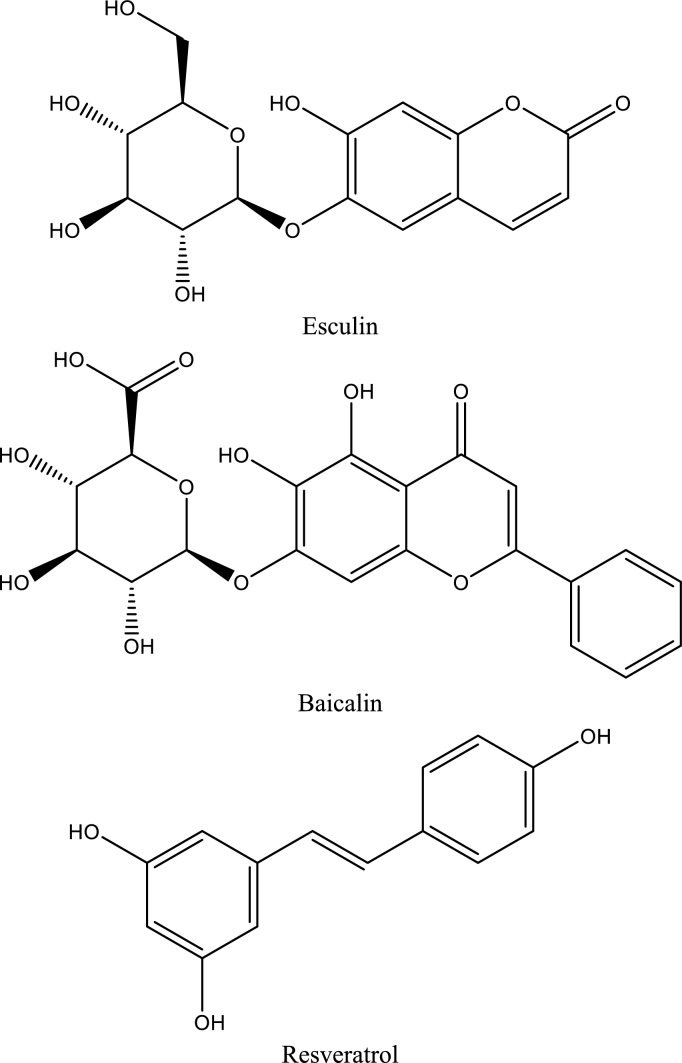

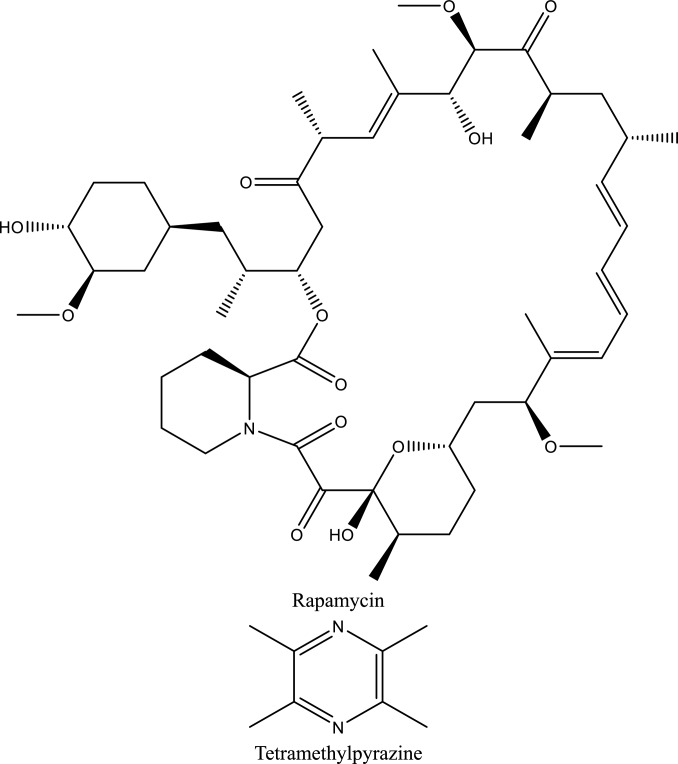

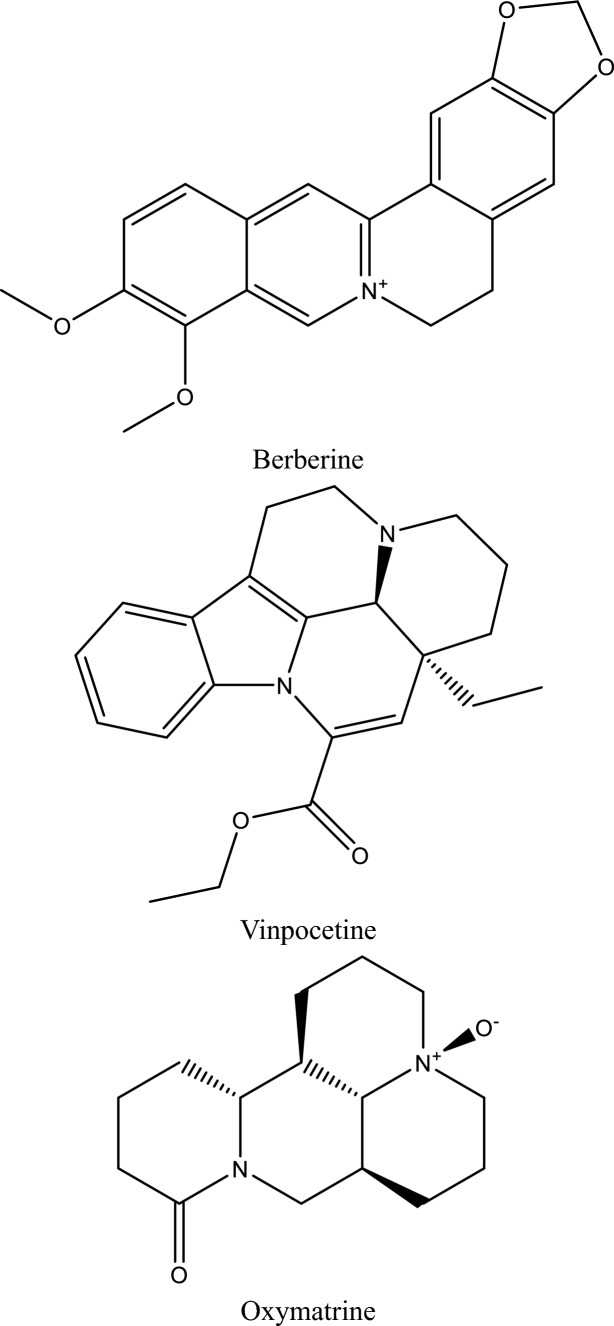

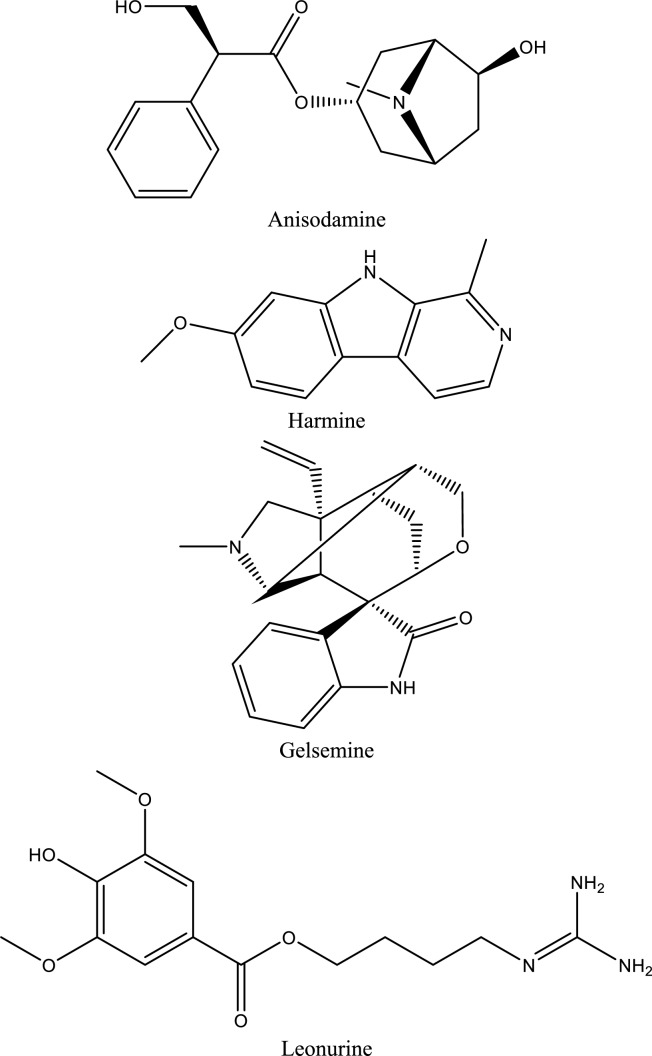

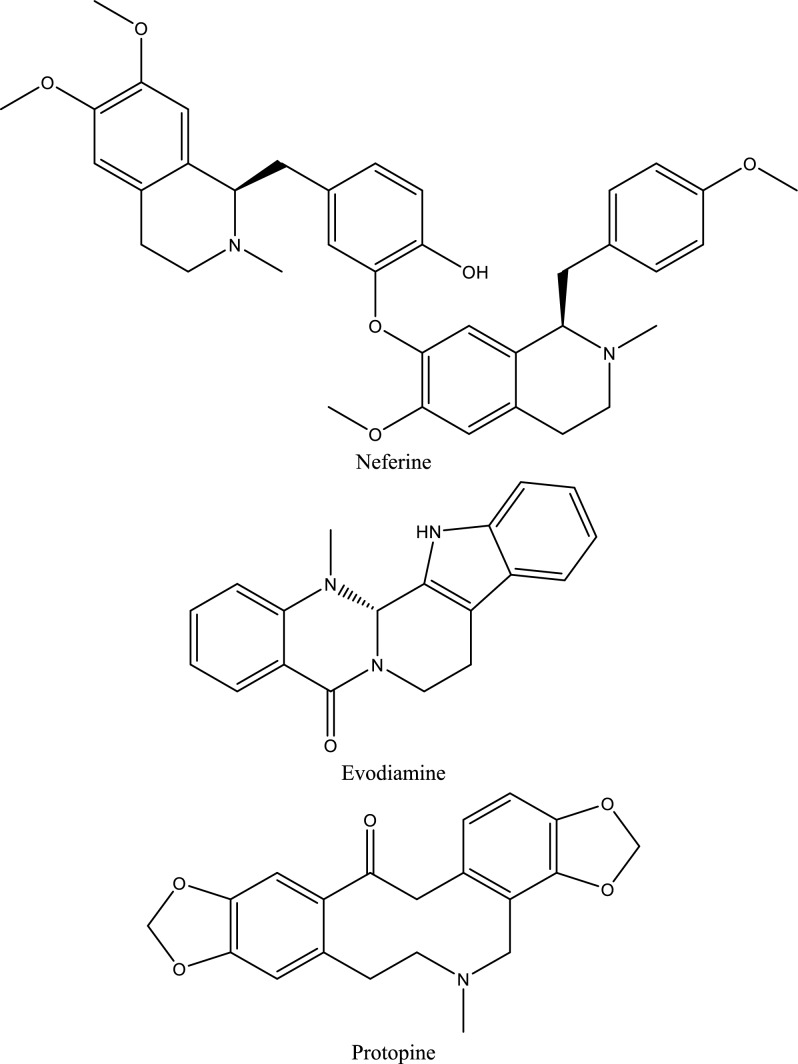

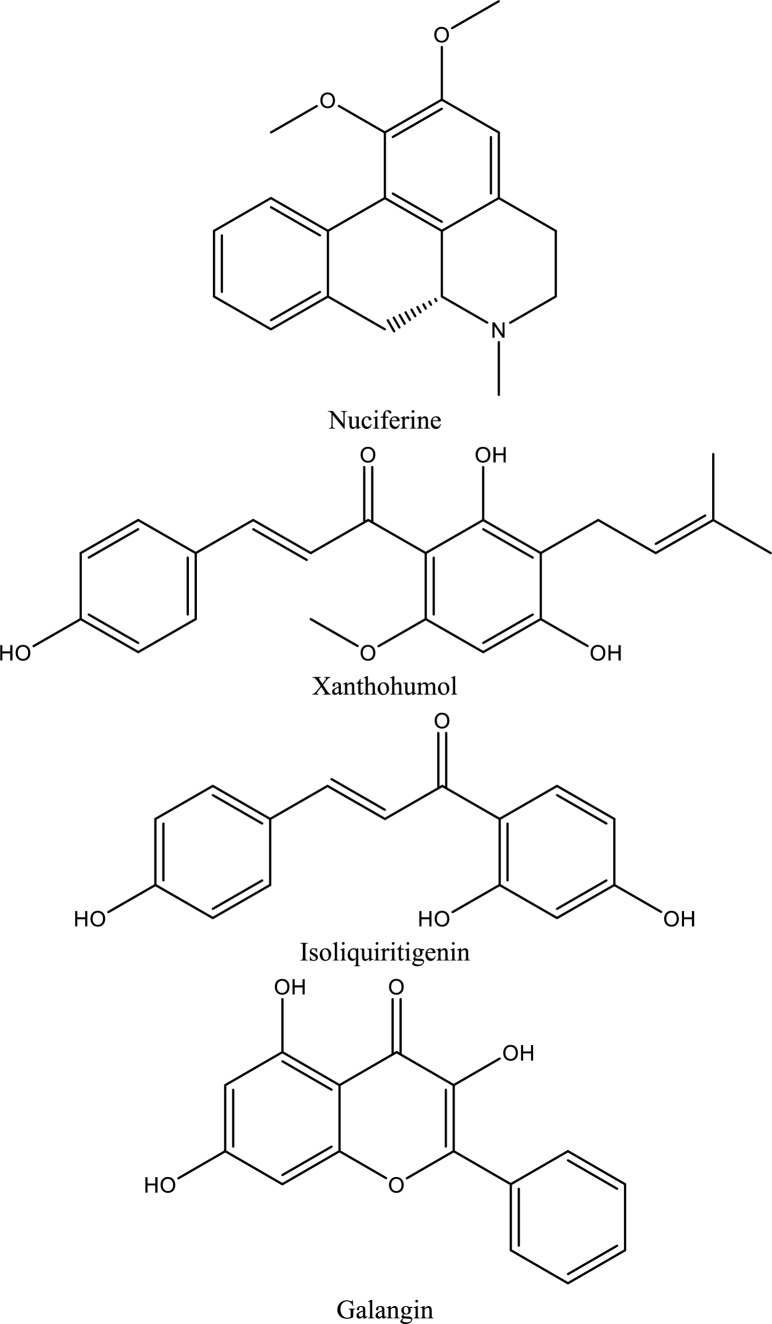

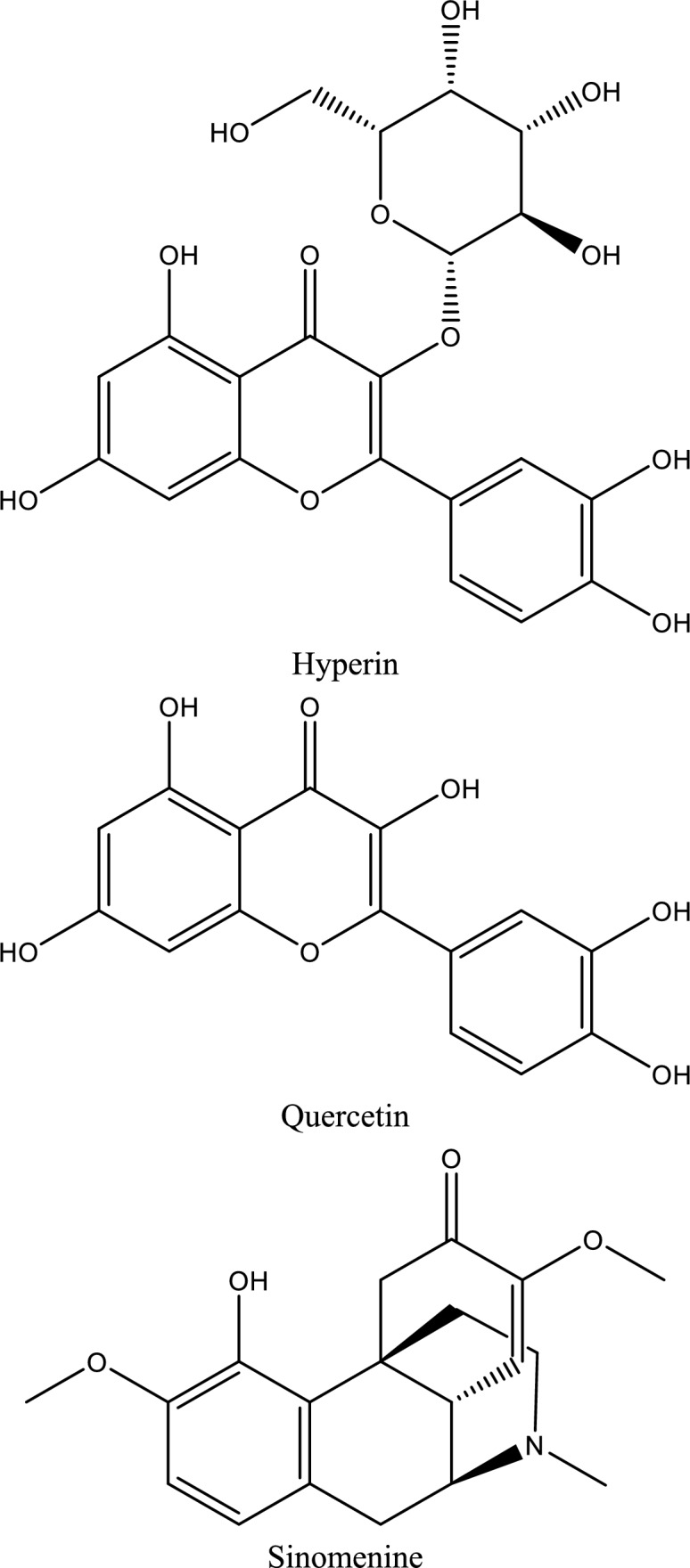

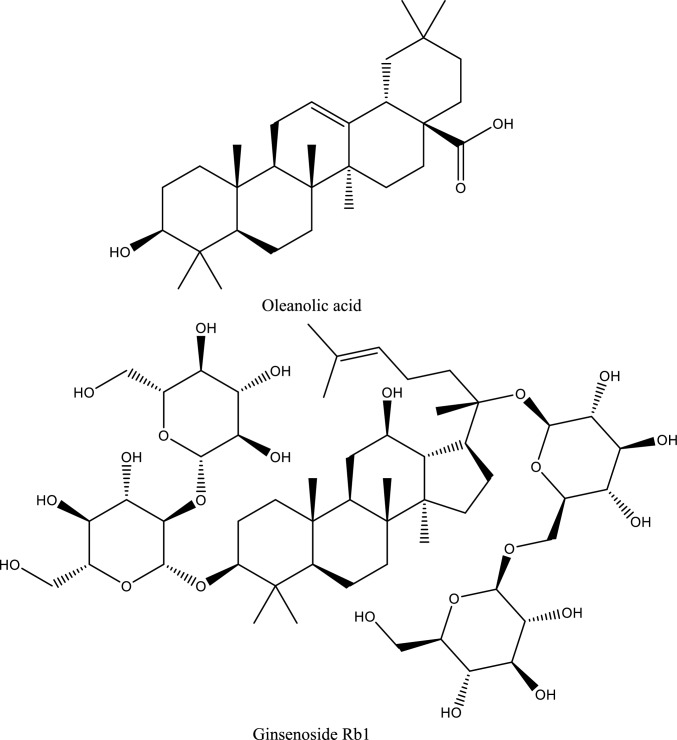

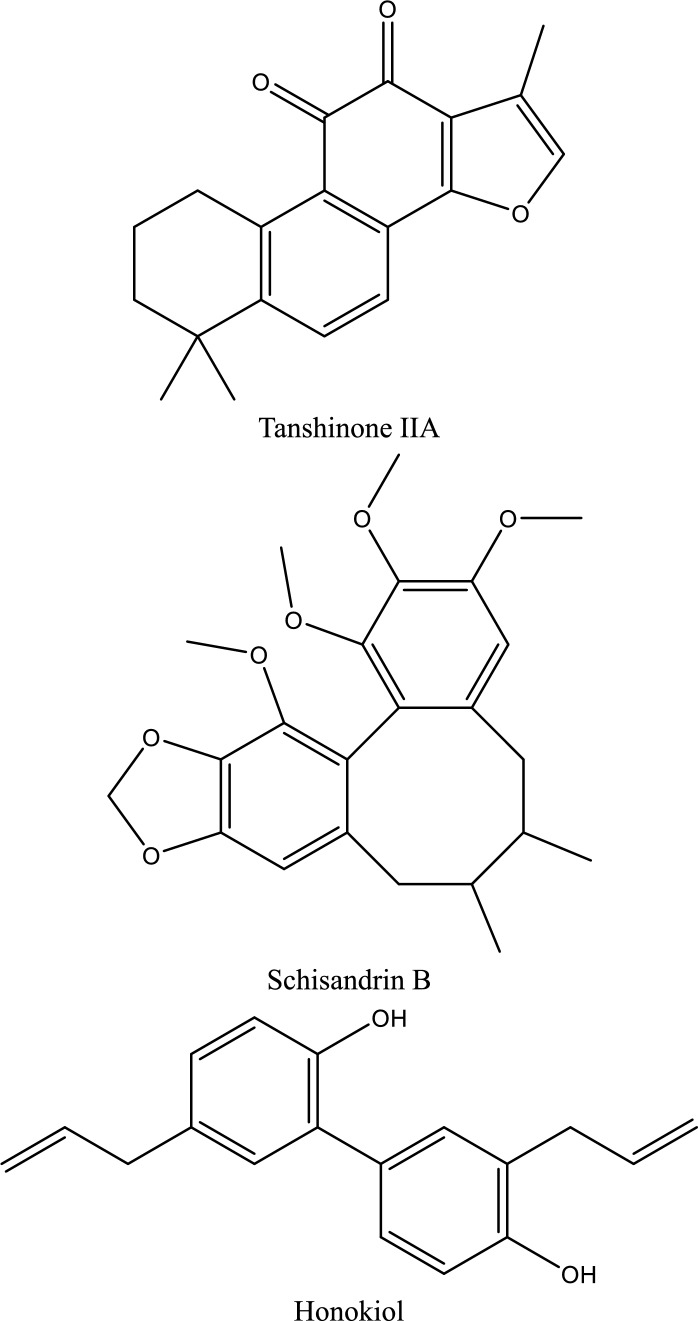

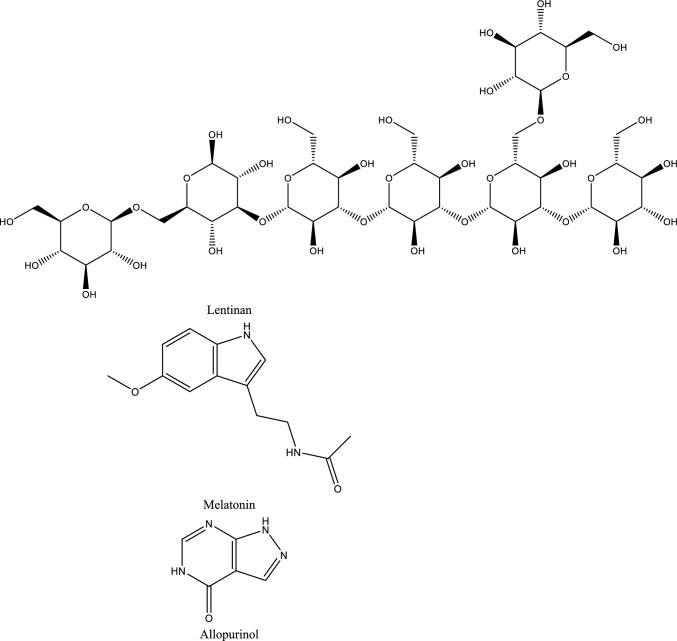

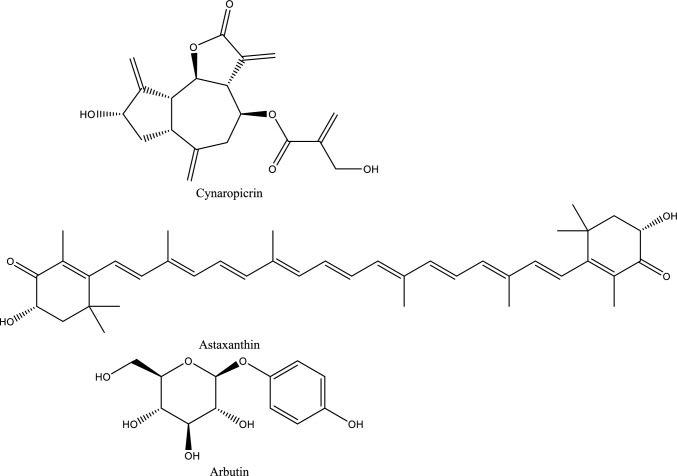

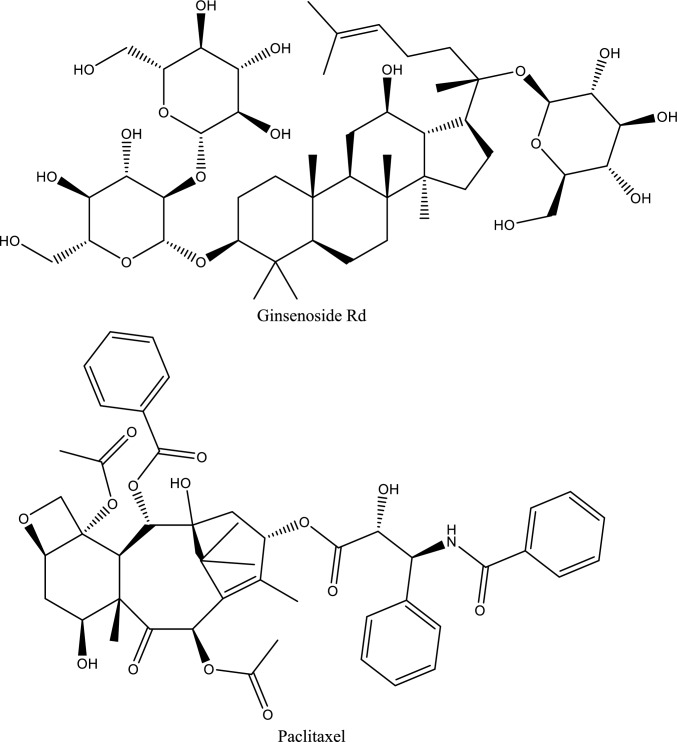

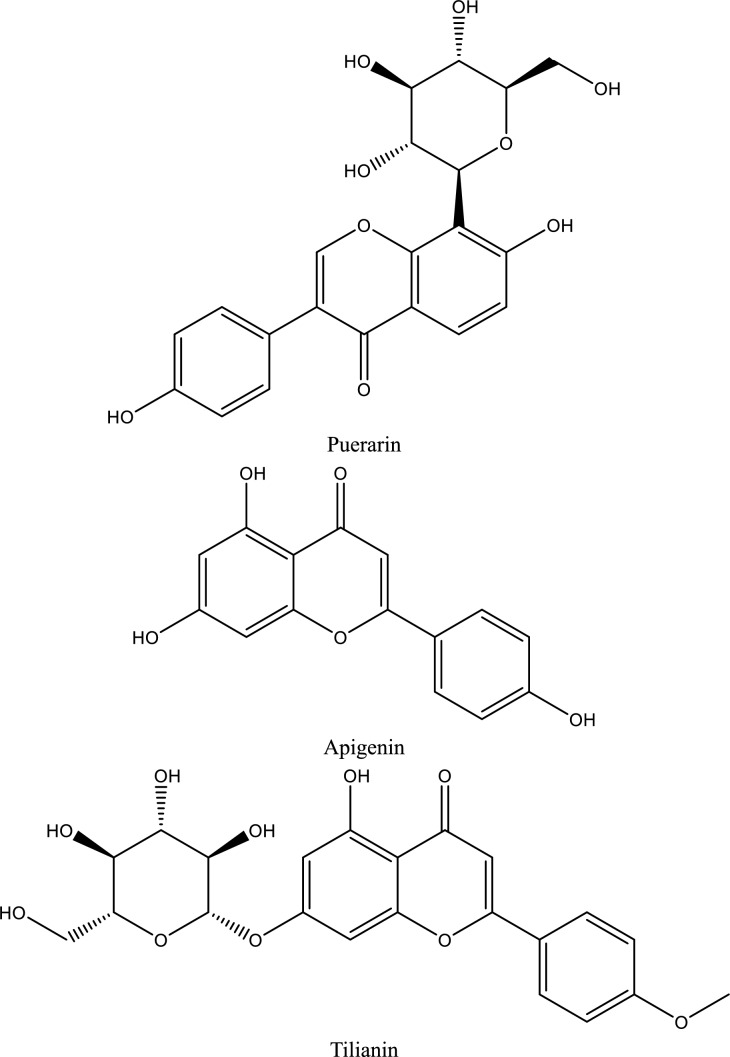

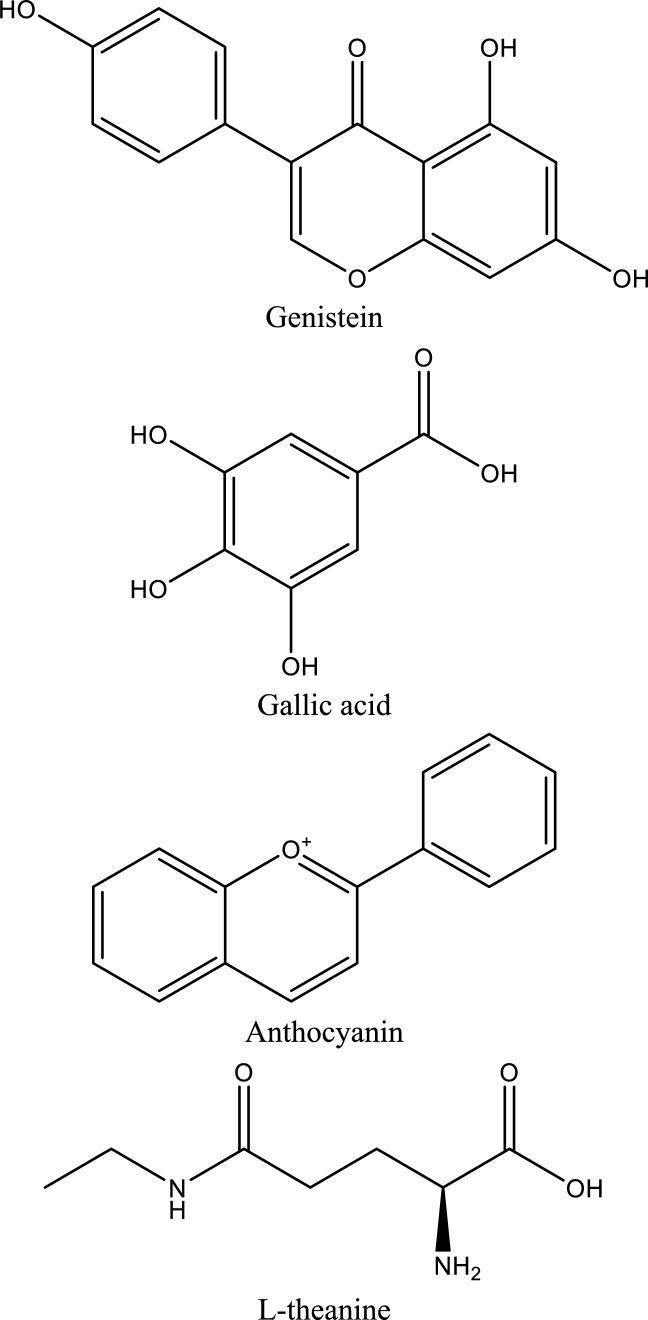

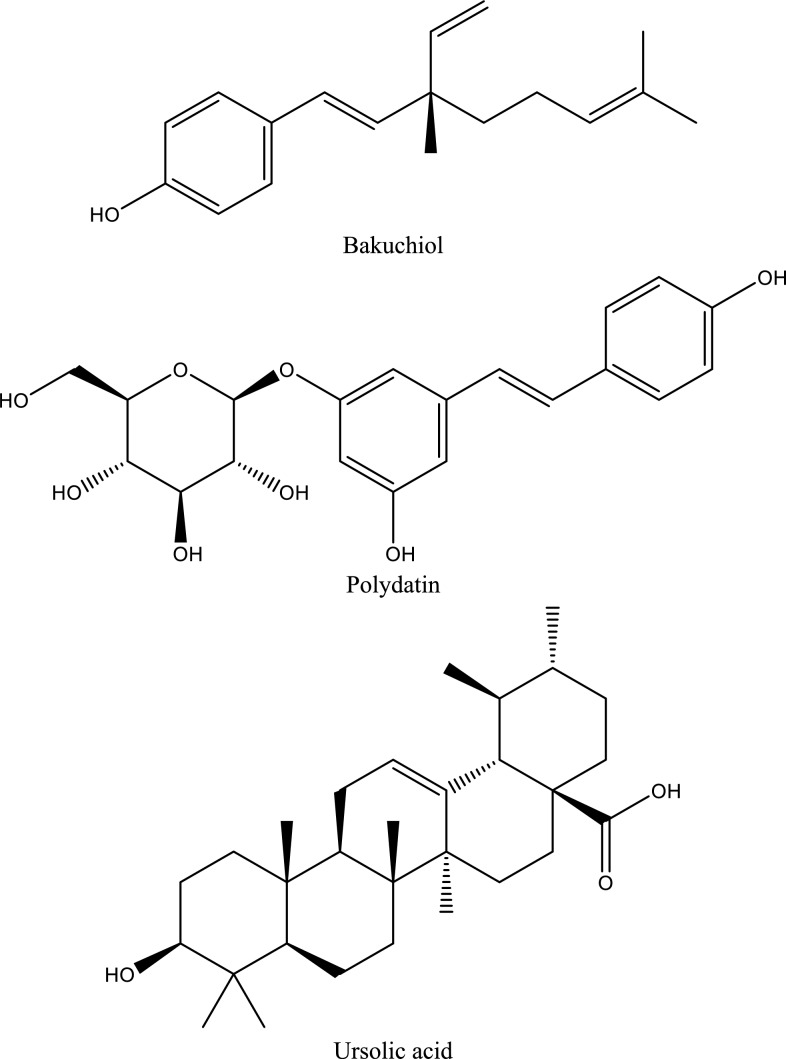

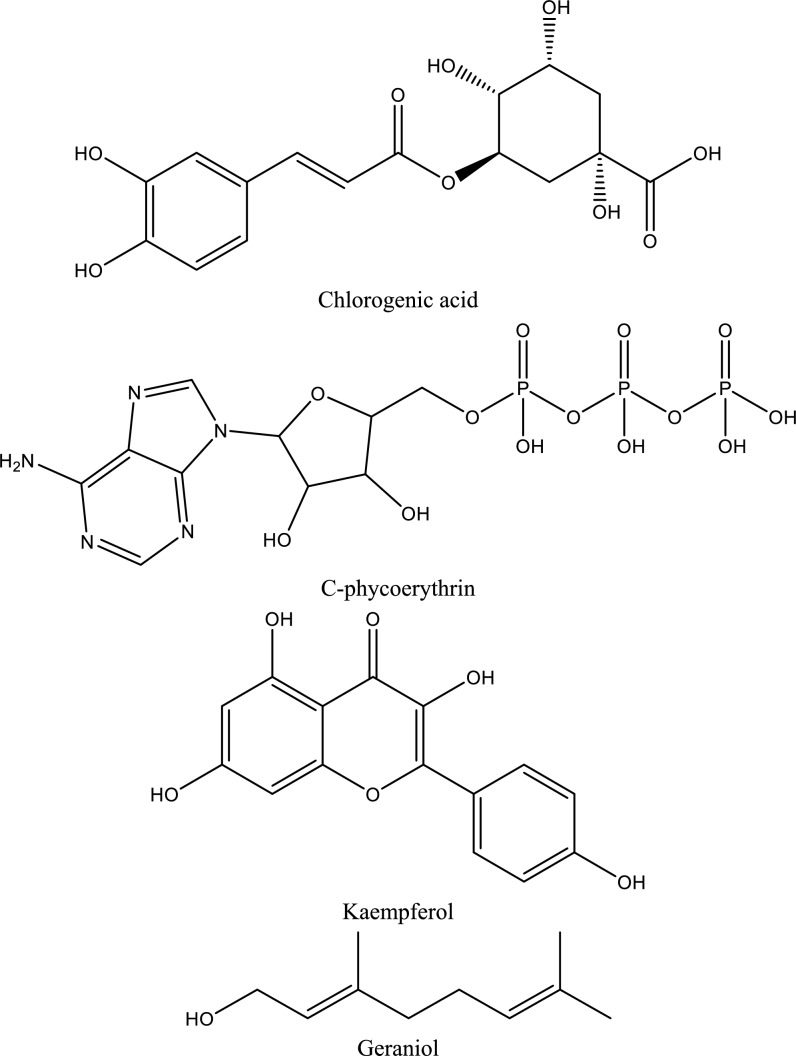

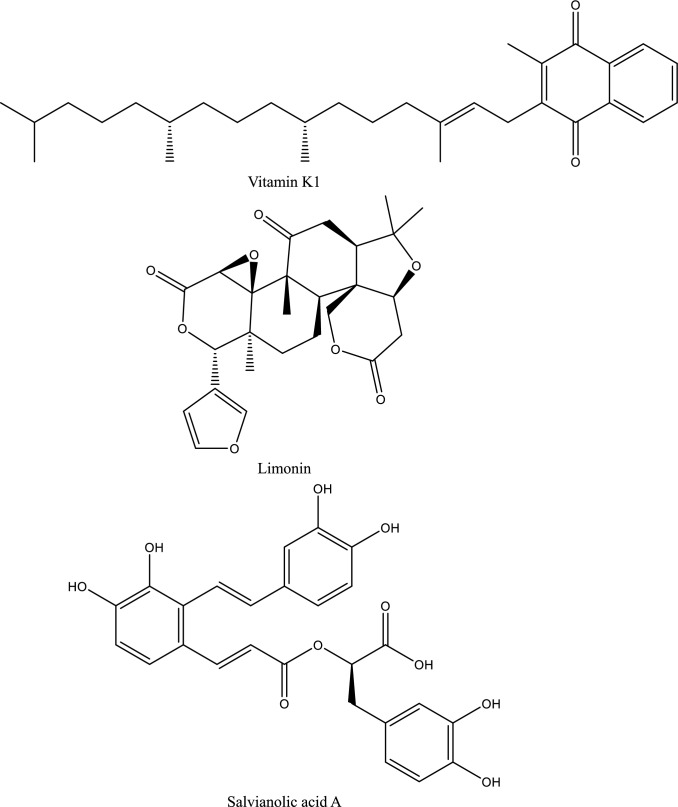

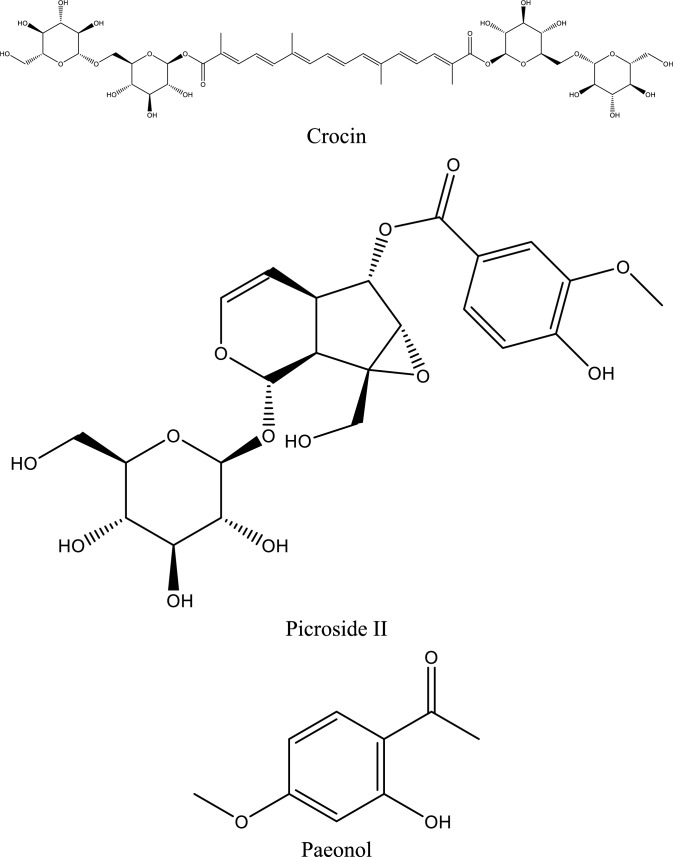

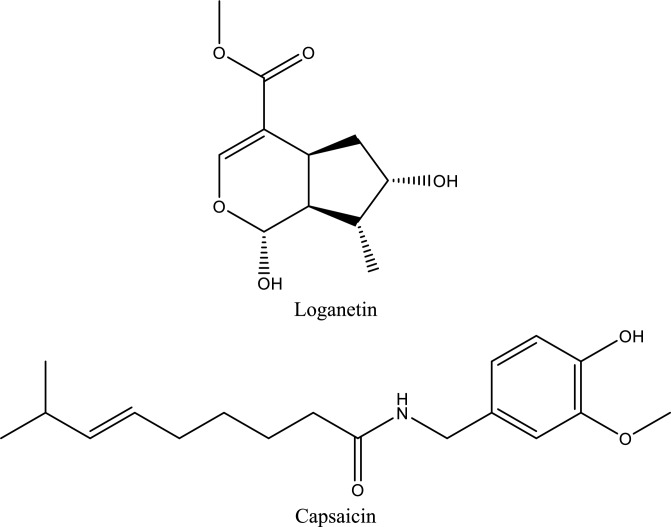
Structures of natural compounds used in the management of AKI

Salvianolic acids (primarily salvianolic acid A [SAA], salvianolic acid B [SAB], and salvianolic acid C [SAC]), polyphenolic constituents of Salvia miltiorrhiza, have been repeatedly reported to mitigate acute kidney injury across chemically induced, ischemic, and inflammatory models by coordinately suppressing oxidative stress, inflammatory signaling, and regulated cell death. In cisplatin- and folic acid–induced AKI, SAB directly bound and activated PRDX5, enhancing redox control and reinforcing the SLC7A11/GPX4 anti-ferroptotic axis to limit lipid peroxidation and tubular cell ferroptosis (Tao et al. 2025). In renal ischemia–reperfusion injury, SAA reduced oxidative stress and apoptosis while promoting prosurvival signaling via Akt/mTOR/4EBP1. It also preserved peritubular capillary endothelial integrity, an important determinant of microvascular inflammation and post-ischemic hypoxia, thereby improving renal function and histologic injury (Song et al. 2018; Zhang et al. 2018c). Consistent with these mechanisms, SAB improved ischemic renal injury by attenuating oxidative stress and inflammatory responses through PI3K/Akt pathway activation (Ma et al. 2017), and in post-contrast tubular injury models, SAB reduced ROS, apoptosis, and inflammatory activation via PI3K/Akt/Nrf2 signaling and by inhibiting the TLR4/NF-κB/NLRP3 axis (Liu et al. 2016; Pei et al. 2022). Beyond classic necroinflammatory injury, SAB also alleviated I/R-associated inflammatory cell death by activating Nrf2 and suppressing caspase-1/GSDMD-mediated pyroptosis and NLRP3 inflammasome signaling (Pang et al. 2020). SAC similarly protected against cisplatin-induced AKI through combined anti-oxidative/anti-inflammatory and anti-apoptotic actions linked to CaMKK–AMPK–Sirt1 signaling (Chien et al. 2021). Collectively, these studies position salvianolic acids as multi-pathway modulators in AKI, with convergent effects on ferroptosis control, TLR4–NF-κB/NLRP3-driven inflammation, Nrf2-centered antioxidant defense, and microvascular/endothelial preservation (Tao et al. 2025; Pang et al. 2020; Song et al. 2018).

Cynaropicrin, evodiamine, and pterostilbene inhibit renal tissue damage in AKI rat models via IL-1β and TNF-α down-regulation (Gao et al. 2018a; Eraslan et al. 2019; Shi et al. 2019; Cai et al. 2021). Nuciferine, a compound from lotus, shows significant kidney protective effects by reducing inflammation, oxidative stress, and fibrosis in models of AKI via inhibition of IL-1β or TNF-α signalling and ferroptosis (Wang et al. 2016, Li et al. 2021a), Genistein, a soy isoflavone, has beneficial effects on a variety of kidney diseases including AKI by regulating key molecular mediators involved in tissue injury, fibrosis, and cellular defense mechanisms. These mediators include TLR-4, MAPK, NF-κB, TGF-β, Smads, ACE, angiotensin, SIRT1, Nrf-2, ROS, SERBP, JAK/STAT, and cytokines (Li et ai. 2017; Gholampour et al. 2020; Katta and Singh 2025).

Collectively, these natural compounds reinforce the concept that effective modulation of the TLR/NF-κB axis in ischemic AKI requires not only suppression of cytokine production but also concurrent inhibition of endothelial CAM expression and leukocyte recruitment. This multi-level regulation may underlie the superior renoprotective efficacy observed with phytochemicals compared to single-target anti-inflammatory agents (Chen et al. 2025).

#### Interference with TLR–adaptor interactions and downstream cytokine release 

Beyond direct inhibition of TLR expression, some natural compounds selectively disrupt TLR–adaptor protein interactions, providing a more targeted modulation of innate immune signaling. At the molecular level, TLR signaling is critically dependent on adaptor proteins, primarily myeloid differentiation primary response 88 (MyD88) and TIR-domain-containing adaptor-inducing interferon-β (TRIF), which determine the specificity, magnitude, and temporal dynamics of downstream inflammatory responses. Dysregulated adaptor recruitment following I/R injury amplifies NF-κB and MAPK signaling, thereby sustaining cytokine release and CAM induction even after resolution of the initial ischemic insult (Akira and Takeda 2004; Kawai and Akira 2010).

Ortho-Vanillin, a simple phenolic compound isolated from Vanilla planifolia and Pinus koraiensis, did not alter TLR2 expression but inhibited the interaction between TLR2 and its adaptor protein MyD88. This disruption reduced NF-κB activation and decreased IL-6 and TNF-α production in LPS-stimulated podocytes and in vivo AKI models (Peng et al. 2019). Importantly, this mode of action highlights a key conceptual distinction between receptor-level blockade and adaptor-level interference. By preserving basal TLR expression while selectively attenuating pathological adaptor recruitment, ortho-vanillin limits excessive inflammatory amplification without globally suppressing innate immune surveillance. This property is particularly advantageous in the context of ischemic AKI, where infection risk remains high (Mulay et al. 2016).

Loganetin, the primary active ingredient of *Cornus officinalis*, ameliorates AKI by inhibiting TLR4 activity and blocking the JNK/p38 pathway (Li et al. 2019c). Given that JNK and p38 MAPKs are downstream effectors of MyD88-dependent TLR signaling, loganetin-mediated suppression of these kinases suggests indirect interference with adaptor-driven signal propagation. This mechanism contributes to reduced transcription of pro-inflammatory cytokines and CAMs, thereby attenuating leukocyte recruitment and microvascular congestion in injured renal tissue (Jang and Rabb 2009; Soares et al. 2019). Additional evidence indicates that selective modulation of TLR–adaptor interactions may also influence the balance between MyD88-dependent pro-inflammatory signaling and TRIF-dependent interferon responses. Natural compounds that bias this adaptor's usage may fine-tune innate immune activation, limiting tissue injury while preserving host defense mechanisms (Kawai and Akira 2010; Chen et al. 2025).

Resveratrol, a stilbene polyphenol found in grapes and berries, has been shown to modulate TLR4 signaling by selectively attenuating MyD88-dependent NF-κB activation while preserving TRIF-dependent interferon responses. This biased adaptor signaling limits excessive pro-inflammatory cytokine release without completely suppressing innate immune defense, a property that is particularly advantageous in ischemic AKI (Youn et al. 2005; Andrade-Oliveira et al. 2015).

Parthenolide, a sesquiterpene lactone derived from Tanacetum parthenium, directly inhibits IκB kinase (IKK) activation downstream of MyD88–IRAK signaling. By targeting adaptor-proximal signaling rather than TLR expression itself, parthenolide suppresses NF-κB–dependent transcription of TNF-α and IL-6 while minimizing off-target immunosuppression (Hehner et al. 1998; Zhang et al. 2014a).

Sulforaphane, an isothiocyanate abundant in cruciferous vegetables, has been extensively reported to exert renoprotective effects in experimental acute kidney injury (AKI) by dual modulation of inflammatory and oxidative stress pathways. Mechanistically, sulforaphane suppresses TLR4-dependent inflammatory signaling, at least in part by inhibiting TLR4 oligomerization and MyD88-mediated downstream activation, leading to reduced NF-κB signaling and decreased expression of endothelial adhesion molecules such as ICAM-1 and VCAM-1 (Youn et al. 2010; Shan et al. 2012). Concurrently, sulforaphane robustly activates Nrf2-dependent cytoprotective programs, including induction of HO-1 and phase II antioxidant enzymes, resulting in attenuation of oxidative stress, preservation of mitochondrial integrity, and improvement of renal microvascular and tubular function in ischemic and inflammatory AKI models (Yoon et al. 2008; Shokeir et al. 2015; Zhao et al. 2016; Liu et al. 2020a, b, c). By coordinating the regulation of TLR4-driven inflammatory signaling and Nrf2-mediated redox homeostasis, sulforaphane effectively limits inflammatory amplification, oxidative injury, and subsequent tissue damage. These processes are tightly coupled in the pathogenesis of ischemic and inflammatory AKI.

Andrographolide, the principal diterpenoid lactone from Andrographis paniculata, has emerged as a pleiotropic immunomodulatory compound with context-dependent effects in renal injury. In experimental models of kidney disease, andrographolide has been shown to attenuate inflammatory signaling and renal tissue damage by suppressing NF-κB– and MAPK-associated transcriptional programs, thereby reducing the expression of pro-inflammatory mediators and limiting tubulointerstitial injury and fibrosis (Ji et al. 2016; Liu et al. 2021). Recent mechanistic studies further extend its renoprotective profile beyond cytokine suppression. In models of sepsis-associated acute kidney injury, andrographolide markedly alleviated tubular injury by inhibiting ferroptosis via activation of the Nrf2/FSP1 axis, leading to reduced lipid peroxidation, preservation of mitochondrial integrity, and improved renal function (Zhang et al. 2024a, b). These findings position andrographolide as a regulator at the intersection of inflammatory signaling, redox homeostasis, and regulated cell death pathways, which are increasingly recognized as central drivers of ischemic and inflammatory AKI progression (Bonventre and Yang 2011). However, accumulating evidence also highlights a potential nephrotoxic liability associated with andrographolide, particularly under conditions of high-dose exposure or unfavorable formulations. Early experimental and clinical observations have documented andrographolide-induced renal tubular apoptosis and autophagy, mediated in part by ROS-dependent endoplasmic reticulum stress and inflammatory signaling in renal epithelial cells (Lu et al. 2014; Gu et al. 2016). More recently, toxicological studies have demonstrated that andrographolide can induce renal tubular injury and cellular senescence by inhibiting SIRT3 signaling, leading to enhanced p53 acetylation and activation of pro-inflammatory and pro-fibrotic transcriptional programs (Cai et al. 2025). Consistent with these mechanistic data, case series analyses have reported acute kidney injury associated with andrographolide-containing preparations, underscoring a dose- and formulation-dependent risk profile (Zhang et al. 2014b). Collectively, these findings underscore that while andrographolide exhibits robust anti-inflammatory and anti-ferroptotic effects in experimental AKI, its dual protective and injurious potential necessitates careful definition of a safe therapeutic window. Optimization of dosing regimens and development of renal-targeted delivery strategies will be essential prerequisites for the clinical translation of andrographolide-based interventions in ischemic and inflammatory kidney diseases.

Collectively, these compounds support the concept that adaptor-level interference represents a refined immunomodulatory strategy capable of uncoupling pathological inflammatory amplification from essential pathogen-sensing functions. Such precision is particularly relevant in ischemic AKI, where excessive inflammation rather than microbial burden is the dominant driver of tissue injury.

#### Glycosides and saponins as regulators of CAM-linked inflammation

CAMs represent critical downstream effectors through which inflammatory signaling pathways translate into leukocyte recruitment, endothelial activation, and microvascular dysfunction in ischemic AKI. Consequently, natural compounds capable of modulating CAM expression occupy a strategic position in interrupting the progression from innate immune activation to tissue-level inflammatory injury.

Among natural products, glycosides and saponins have emerged as particularly effective regulators of CAM-linked inflammation, as they simultaneously suppress upstream inflammatory signaling and directly attenuate endothelial CAM expression. This dual action enables coordinated inhibition of leukocyte adhesion, transmigration, and microvascular congestion within the injured kidney (Bonventre and Yang 2011; Soares et al. 2019).

### Glycosides as modulators of CAM expression

Glycosidic natural products have demonstrated notable efficacy in suppressing inflammatory signaling and CAM expression in ischemic AKI.

Hesperidin, a flavanone glycoside abundant in citrus fruits, has been shown to attenuate renal ischemia–reperfusion (I/R) injury, at least in part, through suppression of the TLR4/NF-κB/iNOS signaling pathway. In a rat model of renal I/R injury, hesperidin treatment significantly reduced renal expression of TLR4, NF-κB p65, iNOS, and caspase-3, accompanied by improvements in inflammatory status, oxidative stress markers, and histopathological injury (Meng et al. 2020).

Similarly, gentiopicroside, a secoiridoid glycoside derived from Gentiana species, protected against LPS-induced AKI by suppressing both MyD88-dependent and TRIF-dependent TLR4 signaling pathways, which induce both type I interferons and inflammatory cytokines (Akira et al. 2006; Kawai and Akira 2010). This was accompanied by reduced renal TNF-α and IL-1β levels, underscoring its capacity to dampen cytokine-driven CAM induction (Shareef and Kathem 2022).

### Saponins as suppressors of CAM-mediated leukocyte recruitment

Saponins represent another major class of natural compounds with potent anti-inflammatory and anti-adhesive properties in ischemic AKI.

Dioscin, a steroidal saponin isolated from Dioscorea species, has been consistently shown to exert renoprotective effects in experimental acute kidney injury, particularly in ischemia–reperfusion (I/R) models. In renal I/R injury, dioscin treatment attenuated tubular and microvascular injury by upregulating heat shock protein 70 (HSP70) expression and suppressing TLR4/MyD88-dependent inflammatory signaling, thereby reducing activation of downstream inflammatory mediators, including cyclooxygenase-2 (COX-2) (Qi et al. 2015). These effects were accompanied by significant downregulation of adhesion molecule expression, such as intercellular adhesion molecule-1 (ICAM-1), attenuation of leukocyte infiltration, and decreased renal production of pro-inflammatory cytokines, thereby limiting inflammatory amplification within the post-ischemic kidney (Qi et al. 2015, 2016). Beyond TLR4/MyD88 inhibition, accumulating evidence indicates that dioscin exerts broader cytoprotective actions by attenuating oxidative stress and regulated cell death pathways, including ferroptosis, necroptosis, and apoptosis, through activation of Nrf2/HO-1 signaling, modulation of SIRT1/SIRT3-associated mitochondrial homeostasis, and suppression of inflammation-driven tubular injury (Zhang et al. 2017a, b, c; Qiao et al. 2018; Wang et al. 2022b, 2024b). Collectively, these findings position dioscin as a multifunctional modulator of innate immune signaling, redox balance, and cell death pathways that are central to the pathogenesis of ischemic and inflammatory AKI.

Hederasaponin C, derived from Pulsatilla chinensis, alleviated sepsis-associated AKI by suppressing TLR4 activation and inhibiting the PIP2/NF-κB/NLRP3 signaling cascade. Importantly, this intervention decreased ICAM-1 expression and reduced endothelial–leukocyte interactions in the renal microvasculature (Shalaby et al. 2023).

Astragaloside IV, a triterpenoid saponin from Astragalus membranaceus, has been widely reported to protect against ischemic and inflammatory renal injury by suppressing NF-κB activation and downregulating CAM expression. Astragaloside IV reduced ICAM-1 and VCAM-1 levels, limited leukocyte adhesion, and improved peritubular capillary perfusion, thereby linking CAM modulation to microcirculatory preservation (Zhang et al. 2018a).

Collectively, glycosides and saponins act as multi-level regulators of CAM-linked inflammation in ischemic AKI. By integrating suppression of upstream inflammatory signaling with direct inhibition of endothelial CAM expression, these compounds effectively disrupt leukocyte recruitment, preserve renal microcirculation, and limit propagation of inflammatory injury (Bonventre and Yang 2011; Chen et al. 2025).

#### Inhibition of endothelial CAM expression by natural compounds

Endothelial activation and subsequent upregulation of cell adhesion molecules (CAMs) constitute a critical downstream event through which upstream inflammatory signaling is translated into leukocyte recruitment, microvascular dysfunction, and tissue injury in ischemic acute kidney injury (AKI). Following I/R, endothelial cells markedly increase the expression of intercellular adhesion molecule-1 (ICAM-1), vascular cell adhesion molecule-1 (VCAM-1), and selectins, thereby facilitating leukocyte rolling, firm adhesion, and transmigration into the renal interstitium. These processes promote capillary obstruction, exacerbate regional hypoxia, particularly in the outer medulla, and amplify tubular epithelial injury (Burne-Taney and Rabb 2003; Bonventre and Yang 2011).

Given this central role, endothelial CAM expression represents a functional convergence point in ischemic AKI pathogenesis. Suppression of CAMs offers a strategy to interrupt inflammatory amplification irrespective of the specific upstream trigger, effectively decoupling inflammatory signaling from tissue-level injury. Natural compounds are uniquely suited to target this downstream effector phase because many simultaneously attenuate inflammatory signaling and directly suppress endothelial CAM expression, thereby reducing leukocyte recruitment and preserving renal microcirculation.

### Suppression of endothelial CAM expression

A growing body of experimental evidence demonstrates that diverse classes of natural compounds inhibit endothelial CAM expression in ischemic and inflammatory AKI models. Polyphenolic flavonoids such as quercetin, luteolin, and epigallocatechin gallate (EGCG) have consistently been shown to downregulate ICAM-1 and VCAM-1 expression in renal endothelial cells. This effect is closely associated with reduced neutrophil and macrophage infiltration and attenuation of microvascular congestion (Liu et al. 2017; Kanlaya and Thongboonkerd 2019).

Luteolin, a flavone widely distributed in medicinal plants, has been shown to reduce renal ICAM-1 and VCAM-1 expression in I/R and endotoxin-induced AKI models. This effect was associated with decreased neutrophil infiltration and preservation of peritubular capillary perfusion, underscoring the role of CAM suppression in microvascular protection (Liu et al. 2017; Siddiqui et al. 2023).

Ferulic acid, a phenolic compound abundant in cereals and medicinal herbs, attenuated ischemic renal injury by inhibiting NF-κB–dependent CAM transcription in endothelial cells. Ferulic acid treatment reduced ICAM-1 expression, improved renal microcirculatory flow, and alleviated hypoxia-driven tubular damage, highlighting its direct relevance to downstream inflammatory effector mechanisms (Zhou et al. 2018; Evans et al. 2013).

Arctigenin, a lignan isolated from Arctium lappa, further exemplifies CAM-centered renoprotection. In renal I/R injury models, arctigenin markedly suppressed ICAM-1 expression and reduced neutrophil and macrophage infiltration, thereby attenuating tubular necrosis and improving renal function (Han et al. 2018).

Epigallocatechin gallate (EGCG), the major catechin in green tea, has consistently demonstrated endothelial-protective effects in AKI by downregulating ICAM-1 and VCAM-1 expression and inhibiting leukocyte adhesion. These effects translated into reduced inflammatory cell accumulation and improved microvascular integrity in experimental models (Kanlaya and Thongboonkerd 2019; Ozer Şehirli et al. 2009).

Pycnogenol®, a standardized extract from Pinus pinaster bark, has been reported to suppress endothelial CAM expression and reduce leukocyte adhesion while preserving capillary integrity. Given its established safety profile and vascular benefits, pycnogenol provides a translationally relevant example of downstream CAM-targeted anti-inflammatory therapy (Ozer Şehirli et al. 2009; D’Amelio et al. 2020).

Glycosidic natural products further exemplify CAM-centered renoprotection. Hesperidin suppresses TLR4/NF-κB/iNOS signaling, leading to reduced ICAM-1 expression and diminished inflammatory infiltration in renal I/R injury (Meng et al. 2020). Gentiopicroside, by inhibiting both MyD88-dependent and TRIF-dependent TLR4 signaling, reduces cytokine-driven CAM induction and limits endothelial activation (Shareef and Kathem 2022). Similarly, loganin and salidroside have been reported to suppress NF-κB–dependent transcription of ICAM-1 and VCAM-1, thereby preserving endothelial barrier integrity and improving renal microcirculatory perfusion (Zhang et al. 2017a, b, c).

Among saponins, dioscin represents a well-characterized example of CAM-targeted anti-inflammatory activity. Dioscin upregulates heat shock protein 70 (Hsp70), which, in turn, suppresses TLR4/MyD88 and COX-2 signaling, resulting in marked reductions in ICAM-1 expression and leukocyte adhesion in renal I/R injury (Qi et al. 2015). Other saponins, including and ginsenosides, similarly downregulate endothelial CAM expression and preserve peritubular capillary density, linking CAM inhibition to improved oxygen delivery and tubular survival (Zhang et al. 2018a).

Hederasaponin C, isolated from Pulsatilla chinensis, alleviated sepsis-associated AKI by inhibiting TLR4 activation and suppressing the PIP2/NF-κB/NLRP3 signaling cascade, resulting in reduced ICAM-1 expression and inflammatory infiltration (Shalaby et al. 2023).

C-phycoerythrin, a phycobiliprotein, exhibited a nephroprotective effect on HgCl2-induced AKI by reducing oxidative stress and ER stress (Blas-Valdivia et al. 2021).

### Inhibition of leukocyte recruitment and microvascular protection

By suppressing endothelial CAM expression, natural compounds indirectly impair integrin–CAM interactions that are essential for leukocyte firm adhesion and transmigration. Attenuation of leukocyte recruitment reduces inflammatory cell accumulation within peritubular capillaries and the interstitium, alleviating capillary compression and improving microvascular perfusion (Friedewald and Rabb 2004; Evans et al. 2013).

Importantly, suppression of CAM-mediated leukocyte trafficking is not merely an epiphenomenon of reduced inflammation but a mechanistically distinct intervention point that directly influences renal oxygenation and functional recovery. Experimental studies have consistently demonstrated that reduced leukocyte infiltration correlates with improved glomerular filtration, decreased tubular necrosis, and enhanced post-ischemic repair (Singbartl et al. 2000; Hayashi et al. 2001).

In addition to compounds primarily targeting CAM expression, several natural products exert renoprotective effects by directly limiting leukocyte recruitment and microvascular obstruction in ischemic AKI. These agents attenuate leukocyte rolling, firm adhesion, and transmigration, thereby preserving peritubular capillary patency and renal oxygen delivery.

Luteolin has been shown to significantly reduce neutrophil accumulation within peritubular capillaries following renal I/R injury. This effect was associated with impaired integrin-mediated firm adhesion and improved microvascular perfusion, leading to attenuation of hypoxia-driven tubular damage (Evans et al. 2013; Liu et al. 2017).

Arctigenin, a bioactive lignan from Arctium lappa, markedly suppressed neutrophil and macrophage infiltration in renal I/R models. Reduction of inflammatory cell recruitment was accompanied by improved capillary flow and decreased tubular necrosis, supporting a direct role for leukocyte trafficking inhibition in renal protection (Han et al. 2018).

Epigallocatechin gallate (EGCG), the major catechin of green tea, has been reported to inhibit leukocyte adhesion to activated endothelial cells and reduce inflammatory cell accumulation in experimental AKI. These effects translated into preserved microvascular integrity and improved renal functional recovery (Kanlaya and Thongboonkerd 2019; Ozer Şehirli et al. 2009).

Ferulic acid further exemplifies downstream modulation of leukocyte recruitment. In ischemic AKI models, ferulic acid reduced inflammatory cell infiltration and alleviated capillary congestion, thereby improving renal microcirculatory flow and oxygenation, independent of direct tubular cytoprotection (Zhou et al. 2018; Evans et al. 2013).

Collectively, these findings indicate that suppression of leukocyte recruitment represents a functionally independent mechanism of renoprotection that complements endothelial CAM inhibition. By preserving microvascular patency and limiting inflammatory cell–mediated capillary obstruction, natural compounds that target leukocyte trafficking directly improve renal oxygenation and post-ischemic recovery (Bonventre and Yang 2011).

### CAM inhibition as a therapeutic convergence point

Collectively, inhibition of endothelial CAM expression represents a unifying downstream mechanism through which structurally and pharmacologically diverse natural compounds exert renoprotective effects in ischemic AKI. By targeting this final effector phase, phytochemicals translate upstream modulation of inflammatory signaling into tangible improvements in microvascular integrity, tissue oxygenation, and tubular survival.

This CAM-centered framework provides a strong mechanistic rationale for prioritizing natural compounds that modulate endothelial activation and leukocyte recruitment. Moreover, it supports the development of therapeutic strategies that combine CAM-targeted natural products with advanced delivery systems, such as nanocarriers, to enhance renal vascular targeting and maximize therapeutic efficacy in ischemic AKI.

#### Natural compounds targeting non-TLR inflammatory and survival signaling pathways in ischemic AKI (Fig. 3)

While inhibition of TLR/NF-κB signaling is a major anti-inflammatory mechanism of many natural compounds, ischemic AKI is orchestrated by a broader, highly interconnected network of cytokine-driven, purinergic, stress-response, and survival pathways. Among these, JAK/STAT signaling, purinergic P2X7 receptor activation, heat-shock protein (Hsp) responses, and PI3K/Akt/mTOR signaling play pivotal roles in determining the balance between inflammatory amplification, regulated cell death, and adaptive repair. Accumulating evidence indicates that natural compounds modulate these non-TLR pathways in a complementary and often synergistic manner, thereby extending their renoprotective effects beyond innate immune receptor inhibition.

#### Modulation of JAK/STAT signaling by natural compounds (Fig. 3)

The Janus kinase/signal transducer and activator of transcription (JAK/STAT) pathway functions as a central cytokine-responsive signaling axis in ischemic AKI. Sustained activation of this pathway, particularly IL-6–mediated STAT3 phosphorylation, drives inflammatory amplification, immune cell recruitment, and maladaptive repair processes that contribute to progression toward chronic kidney disease (Peters et al. 1998; Akcay et al. 2009; Correa-Costa et al. 2012).

Several natural compounds have demonstrated the ability to attenuate ischemic renal injury by suppressing JAK/STAT signaling. Ellagic acid, a polyphenolic compound abundant in fruits and nuts, significantly reduced hypoxia-induced oxidative stress, apoptosis, and inflammation in renal tubular cells by inhibiting phosphorylation of JAK1, JAK2, and STAT1 (Liu et al. 2020a). These effects were associated with improved renal histology and functional parameters, supporting a direct role of JAK/STAT inhibition in renoprotection.

Similarly, magnolol, derived from Magnolia officinalis, has been shown to reduce ischemia–reperfusion-induced renal injury by suppressing JAK2/STAT3 phosphorylation, accompanied by reduced inflammatory cytokine production and apoptosis (Tang et al. 2017; Fu et al. 2021). Shikonin, a naphthoquinone isolated from Symphytum officinale, also modulates JAK/STAT signaling while exerting antioxidant and anti-inflammatory effects, further underscoring the relevance of this pathway as a therapeutic target in ischemic AKI (Zhang et al. 2021b; Li et al. 2023). Genistein, a soy isoflavone, has been shown to inhibit JAK/STAT signaling while simultaneously modulating NF-κB and Nrf2 pathways, thereby reducing inflammatory cytokine production and tubular damage in AKI models (Li et al. 2017; Gholampour et al. 2020).

Collectively, these findings position JAK/STAT signaling as a cytokine-driven amplifier of ischemic AKI, a process that structurally diverse natural compounds can effectively attenuate.

#### Targeting purinergic P2X7 receptor-inflammasome axis

Extracellular ATP released from necrotic and stressed tubular cells functions as a potent danger signal in ischemic AKI. Binding of ATP to the purinergic P2X7 receptor (P2X7R) triggers calcium influx, inflammasome activation, and subsequent release of IL-1β and IL-18, thereby reinforcing inflammatory injury and tubular cell death (Turner et al. 2007; Yan et al. 2015).

Natural compounds have shown promising efficacy in suppressing P2X7R-mediated signaling. Esculin, a coumarin derivative isolated from Vachellia farnesiana, attenuated LPS-induced AKI by inhibiting P2X7R activation, leading to reduced NLRP3 inflammasome assembly, decreased cytokine release, and preservation of tubular epithelial integrity (Cheng et al. 2020; El-Maadawy et al. 2022).

Baicalin, a flavonoid extracted from Scutellaria radix, further exemplifies this mechanism by regulating the Panx-1/P2X7 signaling axis and suppressing pyroptosis in hyperuricemic nephropathy, a process mechanistically relevant to ischemic AKI (Fu et al. 2024). In addition, resveratrol has been shown to inhibit ATP-induced P2X7R activation in a dose-dependent manner, thereby limiting calcium influx, inflammasome activation, and inflammatory cell death (Nuka et al. 2018).

Emerging evidence also implicates epigallocatechin gallate (EGCG) in modulating purinergic signaling. EGCG suppresses ATP-mediated inflammatory responses and inflammasome activation, suggesting an additional layer of regulation at the level of danger signals (Kanlaya and Thongboonkerd 2019). These findings highlight purinergic signaling as a crucial inflammatory node downstream of tubular injury and underscore the therapeutic potential of natural compounds in disrupting ATP-driven inflammatory amplification.

#### Heat-shock proteins as stress-responsive modulators of inflammation and cell survival

Heat-shock proteins (Hsps), particularly Hsp70, serve as endogenous cytoprotective chaperones that preserve protein homeostasis, mitochondrial integrity, and cellular survival during ischemic stress (Kampinga and Craig 2010; Borges et al. 2012). In ischemic AKI, induction of Hsp70 represents a critical adaptive response that limits inflammatory signaling and apoptotic cell death.

Experimental studies have demonstrated that upregulation of Hsp70 protects against renal ischemia–reperfusion injury by suppressing NF-κB activation, reducing pro-inflammatory cytokine production, and inhibiting apoptotic signaling (Wang et al. 2011; O’Neill et al. 2014). Notably, some natural compounds exert renoprotective effects by enhancing Hsp expression. **Dioscin**-mediated induction of Hsp70 was shown to inhibit TLR4/MyD88 and COX-2 signaling, leading to reduced ICAM-1 expression and leukocyte infiltration (Qi et al. 2015).

Beyond inflammation, Hsps modulate the balance between apoptosis and autophagy. Hsp70 promotes autophagic flux and suppresses Bax-dependent mitochondrial apoptosis, thereby enhancing tubular cell survival during ischemic stress (Kaushal 2012; Yang et al. 2013). Compounds such as geranylgeranylacetone and celastrol, which induce Hsp expression, further support the concept that pharmacological activation of stress-response pathways represents a viable renoprotective strategy in ischemic AKI.

#### PI3K/Akt/mTOR signaling: balancing survival, inflammation, and autophagy (Fig. 4)

The PI3K/Akt/mTOR pathway represents a central survival signaling axis in ischemic AKI, regulating cell proliferation, apoptosis, oxidative stress responses, and autophagy. Activation of PI3K leads to Akt phosphorylation, which promotes endothelial and epithelial cell survival while suppressing pro-apoptotic signaling (Duronio 2008; Hoff et al. 2019).

Natural compounds have been shown to modulate this pathway in a context-dependent manner. Lipoic acid and erythropoietin exert renoprotective effects against I/R injury by activating PI3K/Akt signaling, thereby reducing oxidative stress and inflammatory cytokine production (Deng et al. 2013; Rong and Xijun 2015). Conversely, inhibition of mTOR signaling by rapamycin enhances autophagy, reduces ROS generation, and limits inflammatory injury, thereby protecting renal tubular cells (Andrade-Oliveira et al. 2015; Chen et al. 2016a,b).

Several natural compounds, including curcumin and resveratrol, fine-tune PI3K/Akt/mTOR signaling rather than simply activating or inhibiting it. By promoting Akt-mediated survival while restraining excessive mTOR activation, these compounds restore the balance between adaptive autophagy and pathological inflammation, facilitating repair and functional recovery in ischemic AKI (Wu et al. 2017; Den Hartogh and Tsiani 2019). Astragaloside IV further exemplifies this dual regulation, enhancing Akt signaling while preserving autophagic homeostasis, thereby reinforcing its role as a multi-pathway modulator in ischemic renal injury.

##### Integrative perspective: converging signaling pathways as therapeutic targets

Taken together, ischemic acute kidney injury (AKI) is governed by a highly interconnected network of inflammatory and survival signaling pathways that extend well beyond canonical TLR/NF-κB activation. Cytokine-driven JAK/STAT signaling, purinergic P2X7 receptor–inflammasome activation, heat-shock protein–mediated stress responses, and PI3K/Akt/mTOR-dependent survival pathways collectively shape the magnitude and persistence of inflammation, the balance between regulated cell death and survival, and the capacity for tubular and microvascular repair.

Importantly, these pathways do not operate in isolation but converge at critical functional nodes, including endothelial activation, CAM expression, leukocyte recruitment, and microcirculatory integrity. Disruption at these convergence points amplifies hypoxia, perpetuates inflammatory injury, and delays renal recovery.

Natural compounds are uniquely positioned to target this integrated signaling network (Table 1 and Fig. 5). Rather than acting as single-pathway inhibitors, many phytochemicals simultaneously modulate multiple upstream signals while converging on shared downstream effectors, thereby fine-tuning inflammatory intensity, stress adaptation, and survival signaling. This capacity for network-level modulation provides a mechanistic explanation for their broad and robust renoprotective effects observed across diverse experimental models of ischemic AKI.

In contrast to synthetic agents designed to block individual molecular targets, natural products exhibit a systems-oriented mode of action that aligns more closely with the multifactorial pathophysiology of ischemic AKI. This multi-node, convergence-focused regulation supports the concept that natural compounds may function not merely as anti-inflammatory agents but as disease-modifying therapies capable of restoring signaling balance, preserving microvascular function, and promoting adaptive renal repair.

### Nanocarrier-based strategies to enhance targeted modulation of signaling pathways in ischemic AKI (Table 2)

Despite the robust renoprotective effects of natural compounds in experimental models of ischemic acute kidney injury (AKI), their clinical translation has been hampered by unfavorable pharmacokinetic properties, including poor aqueous solubility, rapid systemic metabolism, limited oral bioavailability, and insufficient accumulation within injured renal tissue. These limitations are particularly critical in AKI, where therapeutic windows are narrow, disease progression is rapid, and off-target systemic exposure may exacerbate adverse effects.

Nanocarrier-based drug delivery systems have therefore emerged as a promising strategy to overcome these translational barriers. By improving drug stability, prolonging circulation time, and enabling preferential renal accumulation, nanocarriers enhance the bioavailability and therapeutic index of natural compounds. Importantly, the kidney represents a uniquely favorable target for nanomedicine owing to its high blood flow, fenestrated glomerular endothelium, and increased vascular permeability within injured peritubular capillaries during ischemic and inflammatory states.

From a mechanistic perspective, nanocarriers enable spatiotemporal alignment between drug delivery and pathogenic signaling activation, thereby allowing natural compounds to modulate dysregulated pathways within injured renal tissue with greater precision. Targeted delivery enhances local inhibition of inflammatory and survival signaling cascades, including TLR/NF-κB, JAK/STAT, purinergic P2X7 receptor–inflammasome signaling, heat-shock protein responses, and PI3K/Akt/mTOR pathways, while minimizing systemic immunosuppression.

Furthermore, nanocarrier systems can be engineered to preferentially target activated endothelial cells and inflamed microvasculature, thereby converging on key functional nodes, including endothelial activation, CAM expression, and leukocyte recruitment. This convergence is particularly relevant in ischemic AKI, where endothelial dysfunction and microcirculatory failure represent central drivers of sustained hypoxia and tubular injury.

Collectively, nanocarrier-based strategies transform natural compounds from broadly acting bioactive agents into precision modulators of pathogenic signaling networks. By integrating renal targeting with multi-pathway regulation, nanotechnology provides a rational framework for translating the mechanistic advantages of natural products into clinically viable, disease-modifying therapies for ischemic AKI.

#### Nanocarriers targeting oxidative stress and inflammatory signaling

Polymeric and lipid-based nanocarriers have been extensively explored to enhance renal accumulation and therapeutic efficacy of antioxidant and anti-inflammatory natural compounds. Resveratrol-loaded polyursolic acid nanoparticles demonstrated enhanced reactive oxygen species (ROS) scavenging capacity and anti-inflammatory effects in hydrogen peroxide–induced cellular injury models. In vivo administration further improved renal function and reduced the expression of inflammatory cytokines, indicating effective mitigation of oxidative stress–driven injury (Nie et al. 2023). By prolonging systemic circulation and increasing renal tissue retention, these nanocarriers amplified resveratrol-mediated suppression of NF-κB–dependent inflammatory signaling, thereby extending its biological activity within the narrow therapeutic window of ischemic AKI.

Similarly, curcumin encapsulated within CD44-targeted polymeric nanoparticles selectively accumulated in renal tubular epithelial cells following I/R injury. This receptor-guided delivery significantly enhanced curcumin’s ability to modulate PI3K/Akt/mTOR signaling, leading to reduced oxidative stress, attenuation of tubular apoptosis, and improved histological outcomes (Hu et al. 2018). Notably, targeted nanoparticle delivery enabled sustained intracellular exposure to curcumin, thereby facilitating effective regulation of survival and autophagy-related pathways that are otherwise difficult to achieve with free drug administration.

Collectively, these examples illustrate how nanocarrier-based delivery systems transform natural compounds into precision modulators of oxidative stress and inflammatory signaling. By enhancing local drug concentration and temporal persistence within injured renal tissue, nanocarriers enable more effective suppression of redox-sensitive and inflammation-driven pathways, underscoring their translational potential in ischemic AKI therapy.

#### Mitochondria-targeted nanodelivery and autophagy regulation

Given the central role of mitochondrial dysfunction in ischemic acute kidney injury (AKI), mitochondria-targeted nanocarriers represent a promising strategy to restore cellular energy homeostasis and redox balance. Mitochondrial injury not only drives excessive reactive oxygen species (ROS) production but also amplifies inflammatory signaling and disrupts adaptive stress responses, thereby contributing to tubular cell death and impaired renal recovery.

An L-serine–modified lipid micelle nanosystem co-delivering a mitochondria-targeted curcumin derivative (Cur-TPP) and quercetin effectively reduced mitochondrial ROS generation, suppressed NF-κB activation, and enhanced Nrf2-mediated antioxidant responses in cisplatin-induced AKI models (Zhao et al. 2024). By directly localizing antioxidant and anti-inflammatory agents within mitochondria, this nanosystem coordinated modulation of redox-sensitive inflammatory signaling and mitochondrial stress responses, thereby improving tubular cell survival and renal function.

In addition, ROS-responsive curcumin-loaded nanoparticle systems (NPSBG@Cur) have been shown to activate autophagy while dynamically limiting lipid accumulation in AKI. This effect was associated with modulation of mTOR-dependent signaling and restoration of autophagic flux, thereby promoting adaptive cellular repair rather than maladaptive cell death (Guo et al. 2024a, b). Notably, the ROS-responsive design enabled selective drug release within oxidative microenvironments, allowing real-time adjustment of therapeutic activity in response to mitochondrial stress.

Collectively, these studies highlight the therapeutic potential of mitochondria-targeted, stimulus-responsive nanocarriers to fine-tune the interplay among mitochondrial redox homeostasis, inflammatory signaling, and autophagy. By integrating subcellular targeting with dynamic pathway regulation, such nanodelivery systems offer a sophisticated approach to correcting mitochondrial dysfunction and promoting adaptive repair in ischemic AKI.

#### Enzyme-mimetic and immunomodulatory nanoplatforms

Beyond serving as passive drug-delivery vehicles, enzyme-mimetic nanoplatforms (nanozymes) have emerged as active therapeutic agents capable of directly modulating the oxidative and inflammatory microenvironments in ischemic acute kidney injury (AKI). By mimicking endogenous antioxidant enzymes, these nanomaterials provide sustained and catalytic detoxification of reactive oxygen species (ROS), thereby compensating for impaired intrinsic antioxidant defenses during ischemic stress.

Ultra-small copper-based nanozymes exhibiting catalase-, superoxide dismutase-, and glutathione peroxidase–like activities have demonstrated remarkable efficacy in experimental ischemic AKI models. At extremely low doses, these nanozymes effectively reduced oxidative stress and inflammatory injury while maintaining excellent biocompatibility (Liu et al. 2020b). By directly neutralizing excessive ROS, nanozymes indirectly suppressed redox-sensitive NF-κB activation and downstream CAM expression, thereby reducing leukocyte recruitment and preserving microvascular integrity.

In parallel, immunomodulatory nanoplatforms designed to interact with immune cells or inflammatory mediators selectively have been proposed as next-generation therapies for AKI. Rather than broadly suppressing immune responses, these platforms aim to attenuate pathological innate immune activation, such as excessive cytokine release and inflammasome signaling, while preserving essential host defense mechanisms. This selective immunomodulation aligns closely with the multi-pathway and convergence-based therapeutic framework required for effective treatment of ischemic AKI (Qin et al. 2024; Zhang et al. 2025a, b).

Collectively, enzyme-mimetic and immunomodulatory nanoplatforms extend the scope of nanomedicine from targeted delivery to dynamic regulation of oxidative stress and immune balance. By converging on key functional nodes, including endothelial activation, CAM expression, and microvascular inflammation, these advanced nanotechnologies offer a promising avenue for disease-modifying intervention in ischemic AKI.

#### Translational advantages and remaining challenges of nanocarrier systems

Nanocarrier-based therapeutic strategies offer several distinct translational advantages for the treatment of ischemic acute kidney injury (AKI). By improving renal targeting and prolonging local drug retention, nanocarriers enhance therapeutic efficacy while reducing systemic exposure and off-target toxicity. These properties are particularly valuable in AKI, where therapeutic windows are narrow and systemic immunosuppression or oxidative imbalance may exacerbate disease progression.

Importantly, nanocarrier platforms enable the co-delivery of multiple bioactive compounds or the integration of complementary functions, such as antioxidant activity, modulation of anti-inflammatory signaling, and immune regulation, within a single system. This capacity aligns closely with the multifactorial pathophysiology of ischemic AKI, in which inflammatory signaling, oxidative stress, endothelial dysfunction, and microcirculatory failure converge to drive tissue injury. By addressing multiple pathogenic nodes simultaneously, nanocarriers provide a rational alternative to single-target therapies that have shown limited efficacy in clinical settings.

Despite these advantages, several challenges must be overcome before nanocarrier-based therapies can be successfully translated into clinical practice. From a technical perspective, manufacturing complexity, batch-to-batch reproducibility, and large-scale production remain significant obstacles. From a biological standpoint, long-term biocompatibility, potential immunogenicity, and unpredictable interactions with the injured renal microenvironment require thorough evaluation. In addition, regulatory hurdles associated with nanomedicine, including classification, quality control, and safety assessment, pose substantial barriers to clinical approval.

The heterogeneity of AKI etiologies, disease severity, and patient comorbidities further complicates clinical translation. Effective implementation of nanocarrier-based therapies will therefore require careful patient stratification, optimized timing of intervention, and alignment of nanoplatform design with specific pathogenic mechanisms, such as endothelial activation or microvascular inflammation. Addressing these challenges through standardized formulation protocols, robust preclinical models, and mechanism-driven clinical trial design will be critical for realizing the full therapeutic potential of nanocarrier systems in ischemic AKI.

### Integrative synthesis: from signaling networks to precision therapy in ischemic AKI

Ischemic acute kidney injury (AKI) arises from a tightly interconnected network of microvascular dysfunction, innate immune activation, inflammatory signaling, and maladaptive repair. Central signaling pathways, including TLR/NF-κB, JAK/STAT, purinergic P2X7 receptor signaling, heat-shock responses, and PI3K/Akt/mTOR cascades, function as molecular hubs that translate ischemic stress into sustained cellular injury and inflammatory amplification. Within this network, endothelial activation and upregulation of cell adhesion molecules serve as critical downstream effectors that spatially organize leukocyte recruitment, propagate microvascular obstruction, and exacerbate tissue hypoxia.

Natural compounds are uniquely positioned to intervene in this complex pathophysiology by simultaneously targeting multiple nodes across inflammatory, stress-response, and survival pathways. Through coordinated suppression of pathogenic signaling, preservation of endothelial and microcirculatory integrity, and promotion of adaptive repair mechanisms, these agents offer a systems-level mode of renoprotection that contrasts with conventional single-target approaches.

Importantly, nanocarrier-based delivery systems further refine this multi-target potential by enhancing renal specificity, improving bioavailability, and enabling spatiotemporal control of disease-relevant signaling pathways. By converging mechanistic insight with precision delivery, the integration of natural compounds and nanotechnology supports a paradigm shift toward mechanism-based, multi-node therapeutic strategies tailored to the multifactorial nature of ischemic AKI.

## Conclusion

Ischemic AKI is a complex, multifactorial clinical syndrome arising from the convergence of microvascular dysfunction, innate immune activation, inflammatory signaling, and maladaptive tissue repair. Accumulating evidence indicates that AKI is not merely a transient hemodynamic disturbance but a biologically orchestrated process in which dysregulated intracellular signaling pathways translate ischemic stress into sustained renal injury and long-term functional decline.

This review highlights that key signaling hubs, including TLR/NF-κB, JAK/STAT, purinergic P2X7 receptor signaling, heat-shock protein–mediated stress responses, and PI3K/Akt/mTOR pathways, govern the initiation, amplification, and resolution of ischemic AKI. Importantly, these pathways converge on downstream cellular effectors such as cell adhesion molecules (CAMs), which spatially organize leukocyte recruitment, endothelial–epithelial crosstalk, and inflammatory propagation within the renal microvasculature, thereby linking molecular signaling to functional tissue injury.

Natural compounds emerge as particularly attractive therapeutic candidates in this context due to their capacity to simultaneously modulate multiple pathogenic pathways involved in ischemic AKI. Polyphenols, glycosides, saponins, and related phytochemicals exert renoprotective effects by suppressing inflammatory signaling, attenuating CAM expression, preserving microcirculatory integrity, and promoting adaptive repair mechanisms. This multi-target mode of action distinguishes natural products from conventional single-pathway synthetic agents and provides a compelling mechanistic rationale for their therapeutic potential in AKI.

Furthermore, recent advances in nanocarrier-based delivery systems substantially enhance the translational feasibility of natural compound–based therapies. By improving renal targeting, bioavailability, and spatiotemporally controlled modulation of disease-relevant signaling pathways, nanocarriers enable more precise and effective intervention within the narrow therapeutic window characteristic of AKI.

Collectively, integrating signaling network–based understanding with multi-target natural compounds and precision nanodelivery strategies supports a paradigm shift toward mechanism-driven, disease-modifying therapeutic approaches for ischemic AKI. Future progress will depend on rational pathway selection, optimized delivery platforms, and carefully designed translational studies that bridge experimental efficacy with clinical applicability.

## Challenges and future directions

Despite compelling experimental evidence, several critical challenges must be addressed before natural compound–based therapeutic strategies can be successfully translated into clinical practice for ischemic AKI.

### Bioavailability, pharmacokinetics, and target specificity

A major limitation of many natural compounds is their poor oral bioavailability, rapid systemic metabolism, and insufficient accumulation in injured renal tissue. Future research should prioritize advanced formulation strategies, including nanoparticle encapsulation, receptor-guided delivery, and mitochondria-targeted carriers, to optimize pharmacokinetic profiles and achieve therapeutically effective concentrations at sites of renal injury. Integrating delivery strategies with pathway-specific targeting may further enhance efficacy while minimizing off-target effects.

### Standardization and quality control

The therapeutic reproducibility of natural products is frequently compromised by variability in botanical sources, extraction procedures, and phytochemical composition. Establishing standardized extraction protocols, validated quality-control metrics, and reproducible dosing regimens is essential to ensure consistency across experimental platforms and clinical investigations. Such standardization will be a prerequisite for regulatory approval and large-scale clinical application.

### Safety, toxicity, and drug–natural product interactions

Although natural compounds are often perceived as inherently safe, accumulating evidence highlights the necessity for rigorous toxicological assessment. Dose-dependent organ toxicity, long-term safety profiles, and potential interactions with medications commonly administered to AKI patients, such as antibiotics, vasopressors, and nephrotoxic agents, must be systematically evaluated. This is particularly relevant in critically ill populations, where polypharmacy and altered drug metabolism are common.

### Pathway-specific and stage-dependent therapeutic targeting

AKI is a dynamic and heterogeneous process characterized by temporally distinct phases of injury, inflammation, and repair. Future studies should aim to define stage-specific signaling signatures and identify optimal therapeutic windows for pathway modulation. Precision approaches that tailor interventions to disease stage, injury severity, and patient phenotype, potentially guided by biomarkers such as CAM expression or cytokine profiles, are likely to yield superior clinical outcomes.

### Clinical translation and trial design

Robust clinical trials evaluating natural compound–based therapies for ischemic AKI remain limited. Future investigations should incorporate mechanism-driven endpoints, including biomarkers of endothelial activation, inflammatory signaling, and microcirculatory dysfunction, to enable patient stratification and objective assessment of therapeutic response. Biomarker-guided and adaptive trial designs may be particularly valuable in bridging the gap between promising experimental data and evidence-based clinical practice.

## Final perspective

Advancing the treatment of ischemic acute kidney injury (AKI) requires a fundamental shift away from single-target. These symptom-oriented strategies toward integrative, mechanism-driven interventions reflect the complexity of disease pathophysiology. By aligning natural compounds with key pathogenic signaling networks and leveraging nanocarrier technologies to achieve precise, spatiotemporally controlled delivery, a new generation of multi-target therapeutic strategies becomes feasible.

Such approaches have the potential not only to attenuate acute inflammatory and microvascular injury but also to preserve renal repair capacity and to interrupt the transition from AKI to chronic kidney disease. Ultimately, the convergence of systems-level biological insight with precision delivery platforms may redefine therapeutic paradigms in AKI, offering a rational path toward durable renal protection and addressing one of the most pressing unmet needs in modern nephrology.

## Supplementary Information

Below is the link to the electronic supplementary material.

Supplementary file1 (DOCX 79 kb)

## Data Availability

No datasets were generated or analysed during the current study.
