# Supplementary material for: Natural compounds targeting inflammatory signaling and cell adhesion molecules in ischemic acute kidney injury
Source: Arch Pharm Res. 2026 Apr 26;49(5):577–635. doi: 10.1007/s12272-026-01601-4 (PMC13222333; doi:10.1007/s12272-026-01601-4)
Supplement: Supplementary file 1 — Supplementary file1 (DOCX 79 kb) [file 12272_2026_1601_MOESM1_ESM.docx]

**Table S1. Natural compounds targeting the TLR4/NF-κB signaling pathway in ischemic AKI**

| **Compound (Source)** | **Molecular Target(s)** | **Preclinical Model** | **Study Type** | **Therapeutic Effects** | **Ref.** |
| --- | --- | --- | --- | --- | --- |
| Levistolide (*Ligusticum chuanxiong*) | TLR4, NF-κB | Glycerol-induced AKI in mice | In vivo | Suppressed TLR4/NF-κB; ↓ IL-6, TNF-α; reduced tubular necrosis; improved renal function. | (Shi et al. 2024) |
| Hederasaponin C (Pulsatilla chinensis) | TLR4, NF-κB, NLRP3 | Sepsis-induced AKI in mice | In vivo | ↓ TLR4/NF-κB; ↓ IL-6, TNF-α, IL-1β; ameliorated renal inflammation. | (Shalaby et al. 2023) |
| Gentiopicroside (*Gentiana* spp.) | TLR4, MyD88, NF-κB | LPS-induced AKI in mice | In vivo | Blocked TLR4/MyD88; ↓ NF-κB & cytokines; improved histology; ↓ serum creatinine. | (Shareef and Kathem 2022) |
| Camellia oil (oleic acid) | TLR4-MD2 complex | LPS-induced AKI in rats | In vivo | Bound TLR4-MD2; ↓ TNF-α, IL-2, IFN-γ, IL-1β; ↑ IL-10; reduced renal inflammation. | (Zeng et al. 2020) |
| Hesperidin (citrus flavanone) | TLR4, NF-κB, caspase-3, iNOS | Renal I/R in rats | In vivo | ↓ TLR4/NF-κB, caspase-3, iNOS; protection against oxidative and inflammatory damage. | (Meng et al. 2020) |
| Dioscin (saponin) | TLR4, MyD88, COX-2, HSP70 | Renal I/R in rats + HK-2 cells | In vivo + in vitro | ↑ HSP70; inhibited TLR4/MyD88; ↓ cytokines; preserved tubular structure. | (Qi et al. 2015) |
| Curcumin (*Curcuma longa*) | TLR4, NF-κB | Renal I/R and heatstroke AKI in rats | In vivo | ↓ cytokines & oxidative stress; improved survival and renal function. | (Zhu et al. 2020) |
| Luteolin (flavonoid) | NF-κB, cytokines | Renal I/R in mice | In vivo | ↓ TNF-α, IL-6, IL-1β; reduced inflammatory damage. | (Liu et al. 2017b) |
| Ferulic acid (phenolic acid) | NF-κB, ROS | Renal I/R in mice | In vivo | ↓ cytokines & oxidative stress; anti-inflammatory protection. | (Zhou et al. 2018) |
| Pycnogenol (*Pinus bark extract*) | TNF-α, ICAM-1 | Renal I/R in rats | In vivo | ↓ TNF-α & ICAM-1; improved renal histology & function. | (Ozer Şehirli et al. 2009) |
| EGCG (*Green tea*) | NF-κB, cytokines | Renal I/R in mice | In vivo | ↓ pro-inflammatory cytokines; improved renal biomarkers. | (Lv et al. 2015b) |
| Arctigenin (*Arctium lappa*) | NF-κB, cytokines | Renal I/R in mice | In vivo | ↓ IL-6, TNF-α; anti-inflammatory & anti-apoptotic. | (Han et al. 2018b) |

**Table S2. Natural compounds targeting Cell Adhesion Molecules (CAMs).**

| **Compound (Source)** | **Molecular Target(s)** | **Preclinical Model** | **Study Type** | **Therapeutic Effects** | **Ref** |
| --- | --- | --- | --- | --- | --- |
| Luteolin (flavonoid) | ICAM-1, VCAM-1 | Renal I/R in mice | In vivo | ↓ ICAM-1/VCAM-1; reduced leukocyte adhesion; improved renal recovery. | (Liu et al. 2017a) |
| Ferulic acid (phenolic acid) | ICAM-1, VCAM-1 | Renal I/R in mice | In vivo | ↓ adhesion molecules; limited inflammatory infiltration. | (Zhou et al. 2018) |
| Pycnogenol (*Pinus bark extract*) | ICAM-1 | Renal I/R in rats | In vivo | ↓ ICAM-1; improved renal function. | (Ozer Şehirli et al. 2009) |
| Epigallocatechin gallate (*Green tea*) | ICAM-1, VCAM-1 | Renal I/R in mice | In vivo | ↓ adhesion molecule expression; reduced inflammatory recruitment. | (Lv et al. 2015a) |
| Arctigenin (*Arctium lappa*) | ICAM-1 | Renal I/R in mice | In vivo | ↓ ICAM-1; reduced endothelial dysfunction. | (Han et al. 2018b) |

**Table S3. Natural compounds targeting JAK/STAT signaling.**

| **Compound (Source)** | **Molecular Target(s)** | **Preclinical Model** | **Study Type** | **Therapeutic Effects** | **Ref** |
| --- | --- | --- | --- | --- | --- |
| Ellagic acid (fruits, nuts) | JAK1/2, STAT1 | Hypoxia-induced injury in rats | In vivo | ↓ JAK1/2 & STAT1 phosphorylation; ↓ ROS, apoptosis, inflammation. | (Liu et al. 2020) |
| Pectin (citrus polysaccharide) | JAK2, STAT3 | Renal injury in rats | In vivo | Blocked JAK2/STAT3; ↓ hypertrophy markers & stress proteins. | (Li et al. 2021) |
| Magnolol (*Magnolia officinalis*) | JAK2, STAT3 | Renal I/R in rats | In vivo | ↓ JAK2/STAT3 activation; ↓ apoptosis & inflammation; improved renal function. | (Tang et al. 2017) |
| Shikonin (*Symphytum officinale*) | JAK2, STAT3 | Renal fibrosis in rats | In vivo | ↓ JAK2/STAT3 phosphorylation; ↓ collagen; antioxidant protection. | (Peng et al. 2022) |
| Hydroxysafflor Yellow A (*Carthamus tinctorius*) | JAK2, STAT3 | Renal I/R in rats | In vivo | ↓ JAK2/STAT3; ↓ oxidative stress; improved renal histology. | (Bai et al. 2018) |

**Table S4. Natural compounds targeting the P2X7 receptor.**

| **Compound (Source)** | **Molecular Target(s)** | **Preclinical Model** | **Study Type** | **Therapeutic Effects** | **Ref.** |
| --- | --- | --- | --- | --- | --- |
| Esculin (*Vachellia farnesiana*) | P2X7 receptor, NLRP3 inflammasome | LPS-induced AKI in mice | In vivo | Inhibited P2X7R; ↓ inflammasome activation; reduced tubular damage. | (Serralha et al. 2020) |
| Baicalin (*Scutellaria radix*) | P2X7 receptor, Panx-1 | Hyperuricemic nephropathy in mice | In vivo | Modulated Panx-1/P2X7; prevented pyroptosis; improved renal function. | (Fu et al. 2024) |
| Resveratrol (*Grapes, berries*) | P2X7 receptor, ATP signaling | AKI/CKD models in rodents | In vivo | Inhibited P2X7R; ↓ Ca²⁺ influx; ↓ fibrosis & oxidative stress. | (Carrizzo et al. 2013) |

**Table S5. Natural compounds targeting Heat Shock Proteins (Hsps).**

| **Compound (Source)** | **Molecular Target(s)** | **Preclinical Model** | **Study Type** | **Therapeutic Effects** | **Ref** |
| --- | --- | --- | --- | --- | --- |
| Dioscin (saponin) | Hsp70, TLR4, MyD88, COX-2 | Renal I/R in rats + HK-2 cells | In vivo + in vitro | ↑ Hsp70; ↓ TLR4/MyD88; ↓ cytokines; protection against apoptosis. | (Qi et al. 2015) |
| Curcumin (*Curcuma longa*) | Hsp70, NF-κB | Cisplatin- and I/R-induced AKI in rats | In vivo | ↑ Hsp70; promoted autophagy; ↓ apoptosis; improved renal outcomes. | (Zhu et al. 2020) |

**Table S6. Natural compounds targeting PI3K/Akt/mTOR.**

| **Compound (Source)** | **Molecular Target(s)** | **Preclinical Model** | **Study Type** | **Therapeutic Effects** | **Ref** |
| --- | --- | --- | --- | --- | --- |
| Lipoic acid | PI3K, Akt | Renal I/R in rats | In vivo | Activated PI3K/Akt; ↓ oxidative stress & apoptosis; renal protection. | (Zhang and McCullough 2016) |
| Erythropoietin (endogenous peptide) | PI3K, Akt | Renal I/R in rats | In vivo | Activated PI3K/Akt; ↓ cytokines; improved renal repair. | (Rong and Xijun 2015) |
| Rapamycin (macrolide) | mTORC1 | AKI rodent models | In vivo | Inhibited mTORC1; promoted autophagy; ↓ ROS & cytokines. | (Zhu et al. 2024) |

**Table S7. Natural compounds targeting sonic hedgehog (Shh) pathway.**

| **Compound (Source)** | **Molecular Target(s)** | **Preclinical Model** | **Study Type** | **Therapeutic Effects** | **Ref** |
| --- | --- | --- | --- | --- | --- |
| (−)-Epigallocatechin-3-gallate | Shh, Gli1 | AKI/fibrosis rodent models | In vivo | Inhibition ↓ fibrosis & myofibroblast activation; activation ↑ angiogenesis & tubular repair. | (Lv et al. 2015a) |

**Table S8. Clinical trials, toxicity and dosage of natural products targeting AKI.**

| **Compound** | **Clinical Trials/Preclinical Research** | **Clinical Research Progress** | **Dosage** | **Toxicity** | **Reference** |
| --- | --- | --- | --- | --- | --- |
| **Curcumin** | Clinical trial on chronic kidney disease and contrast-induced nephropathy. | Ongoing clinical studies on chronic kidney disease (CKD) and contrast nephropathy, but more human studies are needed to confirm AKI efficacy. | Curcumin has been administered in clinical trials at doses up to 8,000 mg/day without significant toxicity. | At doses up to 8,000 mg/day, curcumin was well-tolerated, with no serious adverse effects reported. | (Gupta et al. 2013; Zeng et al. 2020) |
| **Hesperidin** | Protective effect against renal ischemia-reperfusion injury in rats. | Preclinical evidence supports its potential; further human clinical trials are required for AKI. | In sub-chronic toxicity studies, hesperidin was administered at doses of 250 and 500 mg/kg/day to rats. | At 1,000 mg/kg/day, hesperidin caused significant alterations in body and organ weights, hematology, clinical chemistry, and tissue histopathology. | (Li et al. 2019b)  (Meng et al. 2020) |
| **Camellia Oil (Oleic Acid)** | Protective effects in LPS-induced AKI in rats. | Clinical trials pending, though promising preclinical results suggest AKI benefit. | Camelina oil, rich in polyunsaturated fatty acids, was administered in acute and sub-chronic toxicity studies. | The oil was found to enhance cellular immunity and did not exhibit significant toxicity at the tested doses. | (Varmira et al. 2024)  (Zeng et al. 2020) |
| **Ortho-Vanillin** | Inhibition of TLR2–MyD88 pathway in AKI. | No human clinical trials yet; preclinical data suggests potential for AKI treatment. | Ortho-vanillin was administered at concentrations of 0.5, 1.0, and 2.5 mM to human cell lines in toxicity studies. | The study showed significant toxicity and protein degradation potential of ortho-vanillin in E. coli grpE: luxCDABE cells after 24 hours. | (Matejczyk et al. 2024)  (Peng et al. 2019) |
| **Dioscin** | Attenuation of renal ischemia/reperfusion injury in rats. | Preclinical trials are promising, with no clinical trials yet for AKI. | A 90-day subchronic toxicity study administered dioscin at doses of 300 mg/kg/day to rats. | Slight gastrointestinal tract distension and hemolytic anemia were observed in male rats at this dose. | (Xu TingTing et al. 2012)  (Qi et al. 2015) |
| **EGCG (Epigallocatechin-3-gallate)** | Protective effect in renal ischemia-reperfusion injury in rats. | Ongoing clinical trials in cardiovascular and metabolic disorders; potential for AKI management in humans. | A study administered EGCG at doses ranging from 50 mg to 1,600 mg to healthy volunteers in a single ascending dose study. | No severe toxicity was reported at these doses. | (Ramachandran et al. 2016)  (Lv et al. 2015b) |
| **Arctigenin** | Anti-inflammatory and anti-apoptotic properties in I/R-induced AKI in mice. | No clinical trial yet for AKI, but preclinical evidence is promising. | A 28-day subchronic toxicity study administered arctigenin at doses of 12 mg/kg/day to rats. | At this dose, focal necrosis and lymphocyte infiltration were observed in the heart, kidney, and liver. | (Tan et al. 2018b)  (Liu et al. 2017b) |
| **Levistolide** | Inhibition of TLR4/NF-κB pathway in acute kidney injury in mice. | No clinical trials yet for AKI, but preclinical trials show significant promise. | Levistolide A was administered at concentrations of 10, 20, and 40 µM to human cell lines in toxicity studies. | No significant toxicity was observed at these concentrations. | (Qu et al. 2021)  (Shi et al. 2024) |
| **Gentiopicroside** | Inhibition of TLR4/MyD88/NF-κB pathway in LPS-induced AKI. | Preclinical data available; clinical trials needed for AKI. | A study administered gentiopicroside at doses of 10, 20, and 40 mg/kg to rats. | No significant toxicity was observed at these doses. | (Tan et al. 2018b)  (Shareef and Kathem 2022) |
| **Luteolin** | Modulation of pro-inflammatory cytokines and oxidative stress in I/R-induced AKI. | No clinical trials yet for AKI, but preclinical studies show significant renoprotective effects. | Luteolin was administered at doses of 10, 20, and 40 mg/kg to rats in various studies. | No significant toxicity was observed at these doses. | (Tan et al. 2018b)  (Liu et al. 2017b) |
| **Ferulic Acid** | Renoprotective effects in renal I/R models. | No clinical trials yet for AKI, but preclinical studies are promising. | A study administered ferulic acid at doses of 10, 20, and 40 mg/kg to rats. | No significant toxicity was observed at these doses. | (Tan et al. 2018b)  (Zhou et al. 2018) |
| **Pycnogenol** | Renal protection in I/R injury models. | No clinical trials in AKI yet, but ongoing studies in other areas show potential for renoprotective effects. | A study administered pycnogenol at doses of 50, 100, and 200 mg/day to humans. | No significant toxicity was observed at these doses. | (Feng et al. 2002)  (Shalaby et al. 2023) |
| **Arbutin** | Protection from kidney damage via PI3K/Akt/Nrf2 pathway. | Preclinical studies show potential, but clinical trials for AKI management are lacking. | A study administered arbutin at concentrations of 0.5%, 1.0%, and 2.0% in topical formulations. | No significant toxicity was observed at these concentrations. | (Mishra et al. 2025)  (Zhang et al. 2021) |

**Table S9. The potency or specificity of different natural compounds targeting the same pathway for AKI management.**

| **Compound** | **Specificity (Targeting NF-κB)** | **Therapeutic Effects (In vivo)** | **Toxicity/Side Effects** | **References** |
| --- | --- | --- | --- | --- |
| Curcumin | Highly specific for NF-κB, moderate effect on other pathways | Effective in reducing renal damage in rat AKI model; ↓ cytokines, ↓ oxidative stress | Potency: Protective at 5–10 mg/kg/day; higher doses (e.g., 200 mg/kg) effective but risk toxicity  Safety: Toxicity graded; max tolerated dose defined; sub-acute studies at up to 3x therapeutic  Clinical limitations: Low bioavailability; excessive dosing may cause systemic toxicity | (Dandekar et al. 2010; Lao et al. 2006; Lou et al. 2023; Rauf et al. 2018) |
| Camellia Oil (Oleic Acid) | Modulates NF-κB via TLR4/MD-2 complex | Anti-inflammatory effects in LPS-induced AKI; improved renal histology | Potency: Oleic acid (10–30 mg/kg for 24 hours) demonstrated protective effects against acute kidney injury by reducing inflammation and oxidative stress in the kidneys.  Safety: 10-nitro oleic acid is safe and well-tolerated in phase 1 clinical study.  Toxicity: No toxicity reported in animal studies or clinical phase 1 trial. | (Amssayef et al. 2025; Bhattacharjee et al. 2020; Hansen et al. 2021) |
| Hesperidin | Moderate specificity for NF-κB, some effect on other inflammatory pathways | Protective effect in renal ischemia-reperfusion models; reduced oxidative stress | Safety: No toxic effects on the kidney or major organs at therapeutic doses.  Toxicity: LD50 = 4837.5 mg/kg; adverse effects observed at ≥1000 mg/kg; safe at ≤500 mg/kg.  Potency: Reduces kidney damage, oxidative stress, inflammation, and fibrosis. | (Chen et al. 2024; Tayal et al. 2023) |
| Dioscin | Moderate specificity for NF-κB, targets multiple inflammatory pathways | ↓ IL-1β, IL-6, TNF-α in renal I/R models; HSP70 upregulation | Safety: Potential cytotoxicity to normal renal cells; risk of renal toxicity in some models  Potency: Reduces oxidative stress, inflammation, and cell death; improves renal function | (Lai et al. 2025; Shi et al. 2023) |
| EGCG (Epigallocatechin-3-gallate) | Specific for NF-κB and multiple anti-inflammatory pathways | Protective effects in AKI models; ↓ oxidative stress and cytokine levels | Safety: Safe at 90–300 mg/day (green tea); ≥800 mg/day (capsules) may cause liver toxicity, not directly kidney toxicity.  Toxicity: No kidney toxicity observed in animal studies; caution with high-dose human supplementation.  Potency: Effective in various kidney injuries; acts via antioxidant, anti-inflammatory, and anti-fibrotic mechanisms. | (Chen et al. 2016; Granato et al. 2020; Hassanein et al. 2023) |
| Arctigenin | Targets NF-κB and multiple inflammatory cytokines | Anti-inflammatory and anti-apoptotic effects in I/R-induced AKI in mice | Safety: Moderate oral toxicity, high IV toxicity, safer subcutaneous; NOAEL <6 mg/kg in dogs; predicted low toxicity computationally.  Toxicity: Renal tubular degeneration, apoptosis in kidney tissue at higher doses or prolonged exposure.  Potency: Protective in DN and TIF models; anti-inflammatory, antifibrotic, and cytoprotective effects; dose-dependent aggravation in I/R injury. | (Han et al. 2018a; Li et al. 2019a; Mei et al. 2024) |
| Luteolin | Specific for NF-κB, modulates inflammatory cytokines | Effective in reducing pro-inflammatory cytokines and oxidative stress in I/R models | Safety: No significant toxicity; safe for kidney function.  Toxicity: Protective against nephrotoxicity; no adverse effects observed.  Potency: Strong antioxidant, anti-inflammatory, and anti-apoptotic effects; effective in multiple kidney injury models. | (Tan et al. 2018a; Zhao et al. 2021) |
| Ferulic Acid | Targets NF-κB and oxidative stress pathways | ↓ cytokines, reduced oxidative stress in I/R-induced AKI in mice | Safety/Toxicity: Low toxicity in cell and animal studies; safe at high doses; further testing recommended.  Potency: Protects against AKI and diabetic kidney injury; restores kidney structure and function | (Mir et al. 2018; Niu et al. 2023) |
| Pycnogenol | Specific for NF-κB and adhesion molecules (ICAM-1, VCAM-1) | ↓ TNF-α, ICAM-1; improved renal markers in I/R models | Safety: Safe, low toxicity, recommended dose 100–360 mg/day, no major renal toxicity.  Toxicity: Low toxicity, protective against cytotoxicity in renal cells.  Potency: Protective in renal I/R injury, reduces cell death in high glucose exposure. | (Cicalese et al. 1999; Kim et al. 2011; Majolo et al. 2021) |
| Arbutin | Specific for NF-κB, targets PI3K/Akt/Nrf2 pathway | Renoprotective effects via PI3K/Akt/Nrf2 signaling in animal models | Potency: Reduces inflammation, oxidative stress, and apoptosis in AKI models; improves kidney function.  Safety/Toxicity: Limited data; ongoing studies to assess safety and toxicity.  Clinical Use: Not yet established; further toxicology and pharmacokinetic studies needed. | (Wang et al. 2024; Zhang et al. 2021) |

**References**

Amssayef A, Elbouny H, Soulaimani B, et al. (2025) The protective effect of Argan oil and its main constituents against xenobiotics-induced toxicities. Fitoterapia 180:106325

Bai J, Zhao J, Cui D, et al. (2018) Protective effect of hydroxysafflor yellow A against acute kidney injury via the TLR4/NF-κB signaling pathway. Scientific reports 8(1):9173

Bhattacharjee B, Pal PK, Chattopadhyay A, Bandyopadhyay D (2020) Oleic acid protects against cadmium induced cardiac and hepatic tissue injury in male Wistar rats: A mechanistic study. Life sciences 244:117324

Carrizzo A, Forte M, Damato A, et al. (2013) Antioxidant effects of resveratrol in cardiovascular, cerebral and metabolic diseases. Food and chemical toxicology 61:215-226

Chen J, Du L, Li J, Song H (2016) Epigallocatechin-3-gallate attenuates cadmium-induced chronic renal injury and fibrosis. Food and Chemical Toxicology 96:70-78

Chen L, Lin W, Zhang H, et al. (2024) TRIB3 promotes malignancy of head and neck squamous cell carcinoma via inhibiting ferroptosis. Cell Death & Disease 15(3):178

Cicalese L, Yacoub W, Subbotin V, et al. Pyruvate inhibits the chronic damage which ensues after ischemia/reperfusion injury of kidneys. In: Transplantation proceedings, 1999. vol 31. Elsevier Inc., p 1033

Dandekar P, Dhumal R, Jain R, Tiwari D, Vanage G, Patravale V (2010) Toxicological evaluation of pH-sensitive nanoparticles of curcumin: acute, sub-acute and genotoxicity studies. Food and Chemical Toxicology 48(8-9):2073-2089

Feng W, Wei H, Liu G (2002) Effect of Pycnogenol® on the toxicity of heart, bone marrow and immune organs as induced by antitumor drugs. Phytomedicine 9(5):414-418

Fu W, Liu Z, Wang Y, et al. (2024) Baicalin inhibits monosodium urate crystal‐induced pyroptosis in renal tubular epithelial cell line through Panx‐1/P2X7 pathways: Molecular docking, molecular dynamics, and in vitro experiments. Chemical Biology & Drug Design 103(4):e14522

Granato D, Mocan A, Câmara JS (2020) Is a higher ingestion of phenolic compounds the best dietary strategy? A scientific opinion on the deleterious effects of polyphenols in vivo. Trends in food science & technology 98:162-166

Gupta SC, Patchva S, Aggarwal BB (2013) Therapeutic roles of curcumin: lessons learned from clinical trials. The AAPS journal 15(1):195-218

Han F, Xia X-x, Wang Y-x, et al. (2018a) Arctigenin: a two-edged sword in ischemia/reperfusion induced acute kidney injury. Biomedicine & Pharmacotherapy 103:1127-1136

Han F, Xia XX, Dou M, et al. (2018b) Arctigenin: A two-edged sword in ischemia/reperfusion induced acute kidney injury. Biomedicine & pharmacotherapy = Biomedecine & pharmacotherapie 103:1127-1136 doi:10.1016/j.biopha.2018.04.169

Hansen A, Rahbek L, Sørensen A, et al. (2021) Nitro-fatty acids decrease type I interferons and monocyte chemoattractant protein 1 in ex vivo models of inflammatory arthritis. BMC immunology 22(1):77

Hassanein EH, Ibrahim IM, Abd-Alhameed EK, Sharawi ZW, Jaber FA, Althagafy HS (2023) Nrf2/HO-1 as a therapeutic target in renal fibrosis. Life sciences 334:122209

Kim YJ, Kim YA, Yokozawa T (2011) Pycnogenol modulates apoptosis by suppressing oxidative stress and inflammation in high glucose-treated renal tubular cells. Food and Chemical Toxicology 49(9):2196-2201

Lai Y, Peng Z, He Z, et al. (2025) Dioscin initiates dual roles in bladder cancer progression via miR-195–5p/FASN/SLC3A2 axis-mediated cell death mechanisms. Translational Oncology 61:102534

Lao CD, Ruffin MT, Normolle D, et al. (2006) Dose escalation of a curcuminoid formulation. BMC complementary and alternative medicine 6(1):10

Li J, Lv Y-g, Pan L-h, et al. (2019a) Toxicity study of 28-day subcutaneous injection of Arctigenin in Beagle dogs. Frontiers in Pharmacology 10:1218

Li Y, Kandhare AD, Mukherjee AA, Bodhankar SL (2019b) Acute and sub-chronic oral toxicity studies of hesperidin isolated from orange peel extract in Sprague Dawley rats. Regulatory Toxicology and Pharmacology 105:77-85

Li Y, Zhou W-W, Sun J-H, et al. (2021) Modified citrus pectin prevents isoproterenol-induced cardiac hypertrophy associated with p38 signalling and TLR4/JAK/STAT3 pathway. Biomedicine & Pharmacotherapy 143:112178 doi:<https://doi.org/10.1016/j.biopha.2021.112178>

Liu Q, Liang X, Liang M, Qin R, Qin F, Wang X (2020) Ellagic acid ameliorates renal ischemic-reperfusion injury through NOX4/JAK/STAT signaling pathway. Inflammation 43(1):298-309

Liu Y, Shi B, Li Y, Zhang H (2017a) Protective Effect of Luteolin Against Renal Ischemia/Reperfusion Injury via Modulation of Pro-Inflammatory Cytokines, Oxidative Stress and Apoptosis for Possible Benefit in Kidney Transplant. Medical science monitor : international medical journal of experimental and clinical research 23:5720-5727 doi:10.12659/msm.903253

Liu Y, Shi B, Li Y, Zhang H (2017b) Protective effect of luteolin against renal ischemia/reperfusion injury via modulation of pro-inflammatory cytokines, oxidative stress and apoptosis for possible benefit in kidney transplant. Medical science monitor: international medical journal of experimental and clinical research 23:5720

Lou X, Lu J, Wang C, et al. (2023) Self-oriented ferritin nanocages mitigate iron overload-induced oxidative stress for acute kidney injury. Chemical Engineering Journal 466:143227

Lv J, Feng M, Zhang L, et al. (2015a) Protective effect of epigallocatechin gallate, a major constituent of green tea, against renal ischemia-reperfusion injury in rats. International urology and nephrology 47(8):1429-35 doi:10.1007/s11255-015-1030-0

Lv J, Feng M, Zhang L, et al. (2015b) Protective effect of epigallocatechin gallate, a major constituent of green tea, against renal ischemia–reperfusion injury in rats. International urology and nephrology 47(8):1429-1435

Majolo F, Martins A, Rehfeldt S, Henriques JAP, Contini V, Goettert MI (2021) Approaches for the treatment of neurodegenerative diseases related to natural products. Studies in Natural Products Chemistry 69:1-63

Matejczyk M, Ofman P, Juszczuk-Kubiak E, et al. (2024) Biological effects of vanillic acid, iso-vanillic acid, and orto-vanillic acid as environmental pollutants. Ecotoxicology and Environmental Safety 277:116383

Mei J, Li X, Xu Z, et al. (2024) Acute and chronic toxicity in Sprague–Dawley rats of orally-administered total lignans from Arctii Fructus, a potential therapeutic drug for diabetes. Regulatory Toxicology and Pharmacology 146:105542

Meng X, Wei M, Wang D, et al. (2020) The protective effect of hesperidin against renal ischemia-reperfusion injury involves the TLR-4/NF-κB/iNOS pathway in rats. Physiology international 107(1):82-91

Mir SM, Ravuri HG, Pradhan RK, et al. (2018) Ferulic acid protects lipopolysaccharide-induced acute kidney injury by suppressing inflammatory events and upregulating antioxidant defenses in Balb/c mice. Biomedicine & Pharmacotherapy 100:304-315

Mishra P, Ahsan F, Mahmood T, et al. (2025) Acute and Subacute Toxicity Study of α‐Arbutin: An In Vivo Evidence. Journal of Applied Toxicology

Niu L, Wang L, He X, et al. (2023) Renoprotective effects of ferulic acid mediated by AMPKα1 against lipopolysaccharide-induced damage. International immunopharmacology 115:109703

Ozer Şehirli A, Şener G, Ercan F (2009) Protective Effects of Pycnogenol against Ischemia Reperfusion-Induced Oxidative Renal Injury in Rats. Renal failure 31(8):690-697 doi:10.3109/08860220903085971

Peng Y, Li Y, Li H, Yu J (2022) Shikonin attenuates kidney tubular epithelial cells apoptosis, oxidative stress, and inflammatory response through nicotinamide adenine dinucleotide phosphate oxidase 4/PTEN pathway in acute kidney injury of sepsis model. Drug Development Research 83(5):1111-1124

Peng Y, Liu L, Wang Y, et al. (2019) Treatment with toll-like receptor 2 inhibitor ortho-vanillin alleviates lipopolysaccharide-induced acute kidney injury in mice. Experimental and Therapeutic Medicine 18(6):4829-4837

Qi M, Zheng L, Qi Y, et al. (2015) Dioscin attenuates renal ischemia/reperfusion injury by inhibiting the TLR4/MyD88 signaling pathway via up-regulation of HSP70. Pharmacological research 100:341-352

Qu X, Guan P, Han L, Wang Z, Huang X (2021) Levistolide A Attenuates Alzheimer's Pathology through Activation of the PPARγ Pathway. Neurotherapeutics 18(1):326-339

Ramachandran B, Jayavelu S, Murhekar K, Rajkumar T (2016) Repeated dose studies with pure Epigallocatechin-3-gallate demonstrated dose and route dependant hepatotoxicity with associated dyslipidemia. Toxicology reports 3:336-345

Rauf A, Imran M, Orhan IE, Bawazeer S (2018) Health perspectives of a bioactive compound curcumin: A review. Trends in food science & technology 74:33-45

Rong R, Xijun X (2015) Erythropoietin pretreatment suppresses inflammation by activating the PI3K/Akt signaling pathway in myocardial ischemia-reperfusion injury. Exp Ther Med 10(2):413-418 doi:10.3892/etm.2015.2534

Serralha R, Rodrigues I, Bertolini A, et al. (2020) Esculin reduces P2X7 and reverses mitochondrial dysfunction in the renal cortex of diabetic rats. Life Sciences 254:117787

Shalaby AS, Eid HH, El-Shiekh RA, et al. (2023) Taming food–drug interaction risk: Potential inhibitory effects of citrus juices on cytochrome liver enzymes can safeguard the liver from overdose paracetamol-induced hepatotoxicity. ACS omega 8(29):26444-26457

Shareef M, Kathem H (2022) Gentiopicroside ameliorates lipopolysaccharide-induced acute kidney injury by inhibiting TLR4/NF-κB signaling in mice model. J Pharm Negat Results 13(4):135-145

Shi J, Li S, Yi L, et al. (2024) Levistolide a attenuates acute kidney injury in mice by inhibiting the TLR-4/NF-κB pathway. Drug Design, Development and Therapy:5583-5597

Shi Y, Shi X, Zhao M, Chang M, Ma S, Zhang Y (2023) Ferroptosis: a new mechanism of traditional Chinese medicine compounds for treating acute kidney injury. Biomedicine & Pharmacotherapy 163:114849

Tan X, Liu B, Lu J, et al. (2018a) Dietary luteolin protects against HgCl2-induced renal injury via activation of Nrf2-mediated signaling in rat. Journal of Inorganic Biochemistry 179:24-31

Tan Y-j, Ren Y-s, Gao L, et al. (2018b) 28-Day oral chronic toxicity study of arctigenin in rats. Frontiers in pharmacology 9:1077

Tang C-Y, Lai C-C, Huang P-H, et al. (2017) Magnolol reduces renal ischemia and reperfusion injury via inhibition of apoptosis. The American Journal of Chinese Medicine 45(07):1421-1439

Tayal R, Munjal K, Gauttam VK, Popli P, Khurana L, Choudhary N (2023) Potential role of hesperidin in lifestyle disorders: A scoping review. South African Journal of Botany 161:542-554

Varmira K, Kahrizi D, Sanjari A, et al. (2024) Non-clinical Safety Evaluation of Camelina Oil: Acute and 12-Week Oral Toxicities. Iranian Journal of Pharmaceutical Research: IJPR 23(1):e140666

Wang Q-L, Zhang P-X, Shen R, et al. (2024) Determination of arbutin in vitro and in vivo by LC-MS/MS: Pre-clinical evaluation of natural product arbutin for its early medicinal properties. Journal of Ethnopharmacology 330:118232

Xu TingTing XT, Zhang Shuai ZS, Zheng LingLi ZL, Yin LianHong YL, Xu LiNa XL, Peng JinYong PJ (2012) A 90-day subchronic toxicological assessment of dioscin, a natural steroid saponin, in Sprague-Dawley rats.

Zeng M, Li M, Zhang B, et al. (2020) Camellia oil inhibits oxidative stress and inflammatory response to ameliorate LPS-induced acute kidney injury via downregulation of TLR4-mediated activation of the NF-κB/AP-1/IRF3 and NLRP3 pathways. Journal of Functional Foods 68:103908

Zhang B, Zeng M, Li B, et al. (2021) Arbutin attenuates LPS-induced acute kidney injury by inhibiting inflammation and apoptosis via the PI3K/Akt/Nrf2 pathway. Phytomedicine 82:153466

Zhang J, McCullough PA (2016) Lipoic acid in the prevention of acute kidney injury. Nephron 134(3):133-140

Zhao J, Li L, Wang Z, et al. (2021) Luteolin attenuates cancer cell stemness in PTX-resistant oesophageal cancer cells through mediating SOX2 protein stability. Pharmacological Research 174:105939

Zhou Q, Gong X, Kuang G, et al. (2018) Ferulic acid protected from kidney ischemia reperfusion injury in mice: possible mechanism through increasing adenosine generation via HIF-1α. Inflammation 41(6):2068-2078

Zhu H, Wang X, Wang X, Liu B, Yuan Y, Zuo X (2020) Curcumin attenuates inflammation and cell apoptosis through regulating NF-κB and JAK2/STAT3 signaling pathway against acute kidney injury. Cell Cycle 19(15):1941-1951

Zhu J, Gong Z, Wang X, et al. (2024) mTORC1 and mTORC2 Co-Protect against Cadmium-Induced Renal Tubular Epithelial Cell Apoptosis and Acute Kidney Injury by Regulating Protein Kinase B. Journal of Agricultural and Food Chemistry 72(36):19667-19679
